# Beyond multidrug resistance: Leveraging rare variants with machine and statistical learning models in *Mycobacterium tuberculosis* resistance prediction

**DOI:** 10.1016/j.ebiom.2019.04.016

**Published:** 2019-04-29

**Authors:** Michael L. Chen, Akshith Doddi, Jimmy Royer, Luca Freschi, Marco Schito, Matthew Ezewudo, Isaac S. Kohane, Andrew Beam, Maha Farhat

**Affiliations:** a Department of Biomedical Informatics, Harvard Medical School, Boston, MA, United States of America; b University of Virginia School of Medicine, Charlottesville, VA, United States of America; c Analysis Group Inc., United States of America; d Critical Path Institute, 1730 E River Rd., Tucson, AZ, United States of America; e Department of Epidemiology, Harvard T.H. Chan School of Public Health, Boston, MA, United States of America; f Division of Pulmonary & Critical Care, Massachusetts General Hospital, Boston, MA, United States of America

**Keywords:** *Mycobacterium tuberculosis*, Multidrug-resistance, Extensively drug-resistant tuberculosis, Machine learning, Genome sequencing

## Abstract

**Background:**

The diagnosis of multidrug resistant and extensively drug resistant tuberculosis is a global health priority. Whole genome sequencing of clinical *Mycobacterium tuberculosis* isolates promises to circumvent the long wait times and limited scope of conventional phenotypic antimicrobial susceptibility, but gaps remain for predicting phenotype accurately from genotypic data especially for certain drugs. Our primary aim was to perform an exploration of statistical learning algorithms and genetic predictor sets using a rich dataset to build a high performing and fast predicting model to detect anti-tuberculosis drug resistance.

**Methods:**

We collected targeted or whole genome sequencing and conventional drug resistance phenotyping data from 3601 *Mycobacterium tuberculosis* strains enriched for resistance to first- and second-line drugs, with 1228 multidrug resistant strains. We investigated the utility of (1) rare variants and variants known to be determinants of resistance for at least one drug and (2) machine and statistical learning architectures in predicting phenotypic drug resistance to 10 anti-tuberculosis drugs. Specifically, we investigated multitask and single task wide and deep neural networks, a multilayer perceptron, regularized logistic regression, and random forest classifiers.

**Findings:**

The highest performing machine and statistical learning methods included both rare variants and those known to be causal of resistance for at least one drug. Both simpler L2 penalized regression and complex machine learning models had high predictive performance. The average AUCs for our highest performing model was 0.979 for first-line drugs and 0.936 for second-line drugs during repeated cross-validation. On an independent validation set, the highest performing model showed average AUCs, sensitivities, and specificities, respectively, of 0.937, 87.9%, and 92.7% for first-line drugs and 0.891, 82.0% and 90.1% for second-line drugs. Our method outperforms existing approaches based on direct association, with increased sum of sensitivity and specificity of 11.7% on first line drugs and 3.2% on second line drugs. Our method has higher predictive performance compared to previously reported machine learning models during cross-validation, with higher AUCs for 8 of 10 drugs.

**Interpretation:**

Statistical models, especially those that are trained using both frequent and less frequent variants, significantly improve the accuracy of resistance prediction and hold promise in bringing sequencing technologies closer to the bedside.

**Research in context**

**Evidence before this study**

Multidrug-resistant tuberculosis (MDR-TB) remains a public health challenge globally. Even in the best equipped laboratories, conventional culture and culture based antimicrobial susceptibility testing constitutes a considerable biohazard and requires weeks to months before results are reported due to the slow *in vitro* growth of *Mycobacterium tuberculosis* (MTB)*.* The recent advent of molecular tests for MDR-TB has been met with considerable enthusiasm. However, these tests rely on targeted amplification of a few select antibiotic resistance genes and have been criticized for being limited in the number of drugs tested and in their sensitivity. It has also proved considerably challenging to extend the same testing platforms that are currently commercially available into a comprehensive diagnostic test for the full panel of anti-tuberculosis drugs. An alternative to targeted mutation detection methods is whole genome sequencing, which captures both common and rare mutations that may be involved in drug resistance. Past studies utilizing whole genome sequencing have most commonly attempted resistance prediction associating individual mutations with resistance (direct association). However, direct association has its drawbacks, as some isolates cannot be classified because they harbor ‘indeterminate’ variants, direct association cannot model gene-gene interactions, and the classification performance declines with higher rates of MDR and higher order resistance.

**Added value of this study**

This study is one of the first to explicitly model the effect of rare and non-causal genetic variants in MTB resistance prediction. While some studies have put forth computational methods for resistance prediction, the combined effect of our rich variant dataset and model architecture that included an innovative multitask wide and deep neural net resulted in a prediction performance that significantly exceeds prior published performance. The multitask/multidrug model is the first to our knowledge to share phenotype and genetic variant data across drugs. We validated both this model and an equally performing simpler penalized logistic regression model using a true independent dataset that was separately collected and sequenced with a different MTB lineage distribution. We also examine important features for resistance to each drug and verify that our analysis is not confounded by lineage.

**Implications of all the available evidence**

With improvements in whole-genome sequencing technologies, the cost of sequencing has decreased and allowed for more routine whole genome sequencing. Our study allows researchers and clinicians to directly use whole genome sequencing to predict MTB drug resistance at a high accuracy with a significantly shorter timeframe compared to phenotypic susceptibility testing. Because our methods are able to predict resistance in less than a tenth of a second, the prediction time for MTB using our method is primarily dependent on sequencing turnaround time. In addition, as routine sequencing increases the MTB sequencing data available, our models can be rapidly updated. We expect that as more data are incorporated, the sensitivity and specificity gap in second-line injectable drugs and fluoroquinolones will become smaller.

Alt-text: Unlabelled Box

## 1 Introduction

Tuberculosis (TB) is among the top 10 causes of mortality worldwide with an estimated 10.4 million new incidents of TB in 2015 [1]. The growing use of antibiotics in healthcare has led to increased prevalence of drug resistant bacterial strains [2], and the World Health Organization (WHO) estimates that 4.1% of new *Mycobacterium tuberculosis* (MTB) clinical isolates are multidrug-resistant (MDR) (*i.e.* resistant to rifampicin [RIF] and isoniazid [INH]). Furthermore, approximately 9.5% of MDR cases are extensively drug-resistant (XDR) (*i.e.* resistant to one second-line injectable drug, such as amikacin [AMK], kanamycin [KAN], or capreomycin [CAP], and one fluoroquinolone, such as moxifloxacin [MOXI], or ofloxacin [OFLX]) [1]. The WHO estimates that 48% of MDR-TB and 72% of XDR-TB patients have unfavorable treatment outcomes, including death or treatment failure, citing the lack of MDR-TB detection and treatment as a global health crisis [1].

Diagnosing drug resistance remains a barrier to providing appropriate TB treatment. Due to insufficient resources for building diagnostic laboratories, fewer than half of the countries with a high MDR-TB burden have modern diagnostic capabilities [3]. Even in the best equipped laboratories, conventional culture and culture based antimicrobial susceptibility testing constitutes a considerable biohazard and requires weeks to months before results are reported due to the slow *in vitro* growth of *Mycobacterium tuberculosis* [1]. Molecular diagnostics are now an increasingly common alternative to conventional cultures, and the WHO has endorsed three such molecular tests: the GeneXpert MTB/RIF, a rapid RT-PCR based diagnostic test assay that detects RIF resistance, the Hain line probe assay (LPA) that tests for both RIF and INH resistance, and the Hain MDRTB*sl*, an LPA that tests for resistance to second-line injectable drugs and fluoroquinolones [1]. The LPAs approved by the WHO have moderate sensitivities, ranging from 63.7% to 94.4% for second-line injectable drugs and fluoroquinolones [[4], [5], [6]]. However, current diagnostic approaches face several challenges. First, these methods have limited sensitivity because they rely on a few genetic loci, ranging between 1 and 6 loci per test [6,7]. Second, they do not detect most rare gene variants of the targeted loci, especially insertions, deletions, and variants in promoter regions. This has been a particular challenge for resistance to drugs like pyrazinamide where many individually rare mutations are thought to be causative [8]. Third, current molecular tests only detect resistance to five anti-tuberculosis drugs, notably missing several key first line agents, including pyrazinamide and ethambutol, and over 5 additional agents currently used for treatment. Fourth, they do not account for variables such as genetic background and gene-gene interactions despite good evidence for their contribution to resistance for several drugs including rifampicin, ethambutol and fluoroquinolones from allelic exchange experiments [[9], [10], [11]]. The limited scope of these tests suggests the need for a comprehensive antimicrobial susceptibility test.

An alternative to targeted mutation detection methods is whole genome sequencing, which captures both common and rare mutations that may be involved in drug resistance. Past studies utilizing whole genome sequencing however have most commonly attempted resistance prediction using direct association (DA), *i.e.* splitting genetic variants into two categories using a list of rules, those that are resistance associated and those that are not, and then classifying a given sample as either resistant or susceptible to the drug of interest. Although this approach works well for drugs like isoniazid and rifampicin for which resistance variants are few and each have large effects, the performance for other first line drugs is considerably lower, at a respective sensitivity (Sn) and specificity (Sp) of 86% and 84% for ethambutol, and 76% and 92% for pyrazinamide in the largest study incorporating over 10,000 isolates [8,[12], [13], [14]]. Notably with DA, 10% or more isolates cannot be classified because they harbor ‘indeterminate’ variants, and the classification performance declines with higher rates of MDR and higher order resistance [14]. The prediction from whole genome sequencing (WGS) data for fluoroquinolones also has limited performance using DA reported at Sn of 45.5% and Sp of 100% [8,12,13].

We hypothesize that the limited predictive performance of WGS for anti-tuberculosis drugs other than isoniazid and rifampicin, that define MDR-TB, could be improved using a large dataset enriched for higher order resistance with incorporation of an expanded scope of variants from genome sequencing and using a model capable of incorporating both additive effects and interactions. We present here an assessment of machine and statistical learning models and genetic feature sets to build a fast and clinically applicable model for detecting anti-tuberculosis drug resistance.

## 2 Materials and methods

### 2.1 Data description

#### 2.1.1 Sequence data

The training dataset consisted of 1379 MTB isolates that underwent sequencing using molecular inversion probes that targeted 28 preselected antibiotic resistance genes and promoter regions, with 100 bases flanking both ends of each region [8]. This sequence data was pooled with 2222 additional MTB whole genome sequences curated by the ReSeqTB knowledgebase, which maintains a public data sharing platform (www.reseqtb.org) curating genotypic and phenotypic data of WHO-endorsed *in vitro* diagnostic assays for MTB [15]. Among the total 3601 MTB isolates that underwent targeted or whole genome sequencing, there were 3227 unique patterns of genetic mutation features and phenotypes. The validation dataset of 792 MTB isolates was obtained by pooling additional data from ReSeqTB, without overlap with the training set, and other MTB whole genome sequences and phenotype data curated manually from the following references [[16], [17], [18], [19]]. The validation isolate genetic mutation features and phenotypes did not duplicate any observed in the training dataset.

#### 2.1.2 Antibiotic resistance phenotype data

All isolates included underwent culture based antibiotic susceptibility testing to two or more drugs at WHO approved critical concentrations and met other quality control criteria as detailed in [8]. The pooled phenotype data included resistance status for eleven drugs: first-line drugs (rifampicin, isoniazid, pyrazinamide, and ethambutol); streptomycin; second-line injectable drugs (capreomycin, amikacin, and kanamycin); and fluoroquinolones (ciprofloxacin, moxifloxacin, and ofloxacin). Phenotypic data was classified as resistant, susceptible, or not available.

### 2.2 Variant calling

We used a custom bioinformatics pipeline to clean and filter the raw sequencing reads. We aligned filtered reads to the reference MTB isolate H37Rv using Stampy 1.0.23 [20] and variants were called by Platypus 0.5.2 [21] using default parameters. Genome coverage was assessed using SAMtools 0.1.18 [22] and read mapping taxonomy was assessed using Kraken [23]. Strains with a coverage of <95% at 10× or more in the regions of interest (Table S8) or that had a mapping percentage of <90% to *Mycobacterium tuberculosis* complex were excluded. Further, regions of the remaining genome not covered by 10 regions or more in at least 95% of the isolates were filtered out from the analysis. In the remaining regions, variants were further filtered if they had a quality of <15, purity of <0.4, or did not meet the PASS filter designation by Platypus.

### 2.3 Building the predictor sets of features

Because 1379 of the 3601 of the MTB isolates in the training set underwent targeted sequencing only, we restricted the resistance predictors to variants in the regions targeted in these isolates (Table S8). The targeted loci were determined by an expert panel of tuberculosis resistance researchers based on literature evidence that they were causative of resistance. The details of this are described previously [8]. We assumed for the purposes of this analysis that any variant found in a locus with a causal link to the drug, excluding silent coding variation, can be causal of resistance. Variants found in other loci not causally implicated with resistance to that drug, are labeled non-causal for that drug, even if they were causally implicated for another drug. Since the *eis* and *rpsA* genes and promoters were recently determined to be causative of kanamycin and pyrazinamide resistance respectively [24,25], we added mutations in the *eis* and *rpsA* regions into our set of predictors. For those isolates with missing genotype data, we used a status of 0.5 for the missing mutations.

The features used for prediction consisted of two groups. In the first group, each mutation was considered a predictor and its status was binary (either present or absent). The second group, we created ‘derived’ categories by grouping the rarer mutations (present in <30 isolates) by gene locus (coding, intergenic and putative promoter regions). For each coding region, we split the variants by type into three groups: single nucleotide substitution (SNP), frameshift insertion/deletion, or non-frameshift insertion/deletion. For each non-coding region, we split the variants by type into two groups: insertions/deletion or single nucleotide substitution). We used individual and derived predictors found in at least 30 MTB isolates to make our final set of predictors used to train all models. We also included wide and deep neural network (WDNN) and L2 regularized logistic regression models trained on subsets of the predictor data. The first subset was mutations or derived categories based on preselected genes known to be important to resistance to the particular drug. The second subset was only mutation predictors without the derived features to measure the effect on accuracy (ACC) of these derived features.

### 2.4 Evaluation of MTB isolate diversity

We identified lineage-defining variants as assessed in a 2015 study by Walker et al. [12]. The genetic-lineage similarity between each pair of isolates was computed as the Euclidean distance between the two-corresponding lineage-defining mutation vectors. We applied Ward's method of hierarchical clustering on the resultant distance matrix [26] to group the isolates and displayed the isolate-isolate Euclidean distance matrix based on the lineage-defining variants in a heat map. We used *hclust* in the R stats 3.4.2 package to perform hierarchical clustering. Each group was mapped back to the recognized MTB lineage classification by matching the expected pattern of SNPs in Walker et al. [12].

### 2.5 Machine learning models

Deep neural networks have had measurable success across several areas of biomedicine. Here, we evaluated several neural network architectures for ACC in predicting MDR-TB. These included a multidrug (MD) and single drug (SD) wide and deep neural network (WDNN) [27] and a deep multilayer perceptron (MLP), with the same architecture as MD-WDNN without the ‘wide’ logistic regression model. The SD-WDNN and MLP underwent the same training process as the MD-WDNN (Table S9). We also implemented two simpler machine learning classification models – a single drug random forest and a simple drug L2 regularized logistic regression, to test the relative performances of simpler and more complex machine learning models. See the supplement for the hyperparameters of each model. The WDNN [27], marries two successful models, logistic regression and deep multilayer perceptrons (MLP), to leverage the strengths of each approach. In WDNNs, a ‘wide’ logistic regression model is trained in tandem with a ‘deep’ MLP and the two models are merged in a final classification layer, allowing the network to learn useful rules directly from the input data and higher level nonlinear features. For genomic data, the logistic regression portion of network can be thought of as modeling the additive portion genotype-phenotype relationship, while the MLP models the nonlinear or epistatic portion. We implemented a WDNN with three hidden layers each with 256 rectified linear units (ReLU) [28], dropout [29], batch normalization [30], and L2 regularization (Fig. 7). Dropout and L2 regularization are used to prevent overfitting of the models to the training data. L2 regularization was applied on the wide model (which is equivalent to the well-known Ridge Regression model) [31], the hidden layers of the deep model, and the output sigmoid layer. The network was trained *via* stochastic gradient descent using the Adam optimizer for 100 epochs with randomized initial starting weights determined by Xavier uniform initialization.

The MD-WDNN was trained simultaneously on resistance status for all 11 drugs, including ciprofloxacin. Each of the 11 nodes in the final layer represented one drug and its output value was the probability that the MTB isolate was resistant to the corresponding drug. We constructed a single-drug WDNN (SD-WDNN) with the same architecture as the multidrug model except for the structure of the output layer, which predicts for one drug.

The MD-WDNN utilized a loss function that is a variant of traditional binary cross entropy. Our dataset had a missing resistance status for some drugs in the MTB isolates, so we implemented a loss function that did not penalize the model for its prediction on drug-isolate pairs for which we did not have phenotypic data. Due to imbalance between the susceptible and resistant classes within each drug, we adjusted our loss function to upweight the sparser class according to the susceptible-resistant ratio within each drug. Thus, the final loss function was a class-weight binary cross entropy that masked outputs where the resistance status was missing.

### 2.6 Training and model evaluation

To assess each model, we performed ten-fold cross-validation, repeated 5 times, for a total of 50 different validation sets. The metric used for evaluation was the area under the receiver-operator curve (AUC) and overlap of its 95% confidence interval between the models. All single drug models were stratified by class label to address imbalances between resistance and susceptible classes, as they were all single task classifiers. The highest performing simple and complex models, L2 regularized logistic regression and the MD-WDNN trained on the full predictor set, were validated through an independent validation set.

For the MD-WDNN and L2 regularized logistic regression on the full predictor set, we reported two pairs of Sn and Sp performance on the independent validation set. We reported the first pair by determining a probability threshold to maximize the sum of Sn and Sp for each drug. For the second pair, we determined the probability threshold to maximize Sn given that the Sp is at least 90%. The 90% specificity threshold stems from the value assessment that over-diagnosis of antibiotic resistance is more harmful than under-diagnosis due the treatment toxicity and side effects, *e.g.* renal failure and hearing loss, for the drugs used in antibiotic resistant cases.

Hyperparameters were determined using Bayesian optimization as implemented by Spearmint [32]. We reported the prediction time for the MD-WDNN and L2 regularized logistic regression on the independent validation set, averaged over 1000 iterations.

### 2.7 MTB isolate visualization using t-SNE

We applied *t-*distributed Stochastic Neighbor Embedding (*t-*SNE), a method for visualizing data with high dimensionality [33], to two datasets: (1) the set of input predictors (common mutations and derived categories) and (2) the final output layer of the MD-WDNN. The genetic markers, originally in 222 dimensions, and the final layer weights, originally in 11 dimensions, were each extracted from the MD-WDNN and projected onto two dimensions. Each point represented one MTB isolate and was colored based on its phenotypic status for each drug. The lineage clustering was also overlaid on the *t*-SNE plots to determine the effect of lineage on the two different representations of isolates.

### 2.8 Importance of MTB genetic variants to drug resistance

We examined predictor importance to resistance by analyzing the prediction outputs of the MD-WDNN and the presence or absence of mutations through a permutation test. We permuted the resistance labels and calculated the distribution of following difference:

$$\begin{array}{c} P\left(\mathrm{isolate is resistant}\;\right|\;\mathrm{mutation is present})\\ -P\left(\mathrm{isolate is resistant}\;\right|\;\mathrm{mutation is absent}) \end{array}$$

where P(isolate is resistant | mutation is present) is the MD-WDNN's probability of resistance for a given mutation. We then compared the actual differences with the permuted differences. The sampling distribution included 100,000 randomized permutations per mutation and the actual differences were evaluated at a significance level of α = 0.05 using a Bonferroni correction for the 222 multiple comparisons. We conducted the permutation test for each predictor (common mutations or derived categories) that was present in at least 30 MTB isolates.

We also examined predictor importance to the L2 regularized logistic regression model. Using bootstrapping with 10,000 iterations, we built a confidence interval for each predictor-drug pair's exponentiated β coefficient (commonly referred to as the odds-ratio) to determine resistance or susceptibility importance. The confidence interval was built at a significance level of α = 0.05 using a Bonferroni correction for the 222 multiple comparisons.

### 2.9 Implementation details

All WDNN and MLP model implementations used the Keras 1.2.0 library in Python 2.7 with a TensorFlow 0.10.0 backend. The random forest and regularized logistic regression classifiers were implemented with Python Scikit-Learn 0.18.1. The isolate diversity analysis was implemented using R 3.4.0, the *t*-SNE analysis used the Rtsne 0.13 package in R, and the permutation tests were implemented in Python 2.7. All models were trained on a NVIDIA GeForce GTX Titan X graphics processing unit (GPU). Hyperparameters are available in Table S9. All analysis code and input data files are openly available at https://github.com/farhat-lab/wdnn.

## 3 Results

### 3.1 Data Processing

The pooled data from the WHO network of supranational reference laboratories and the ReSeqTB knowledgebase [8,15] used in training the initial model included 3601 MTB isolates. Ofloxacin was tested on the smallest number of isolates at a total of 739. All other drugs were tested in at least 1204 isolates, with rifampicin tested in 3542 isolates and isoniazid in 3564 isolates (Table S1). A high proportion of isolates tested resistant, ranging from 19.9% to 47% for the different drugs.

The independent validation set contained 792 MTB isolates, with 198 to 736 of these isolates tested for each of the 10 drugs (Table S2). Because ciprofloxacin had limited phenotypic availability in the independent validation set and predictive performance could not be validated, we did not include performance for ciprofloxacin resistance.

We found 6342 different insertions, deletions, and single nucleotide polymorphisms (SNPs) in 30 promoter, intergenic, and coding regions of the MTB isolates' genomes. Of these variants, 166 were present in at least 30 of the 3601 isolates and were used as predictors in the “Common Mutations” predictor set. Of the 3445 variants found in fewer than 30 isolates, we aggregated the variants into 141 derived categories (see Methods) and used 56 of them, those present in at least 30 isolates, as predictors in the “Common Mutations” and rare aggregate variants predictor set. The final model used these 222 total predictors in training and subsequent analyses.

### 3.2 Evaluation of MTB isolate diversity

**Fig. 1:**
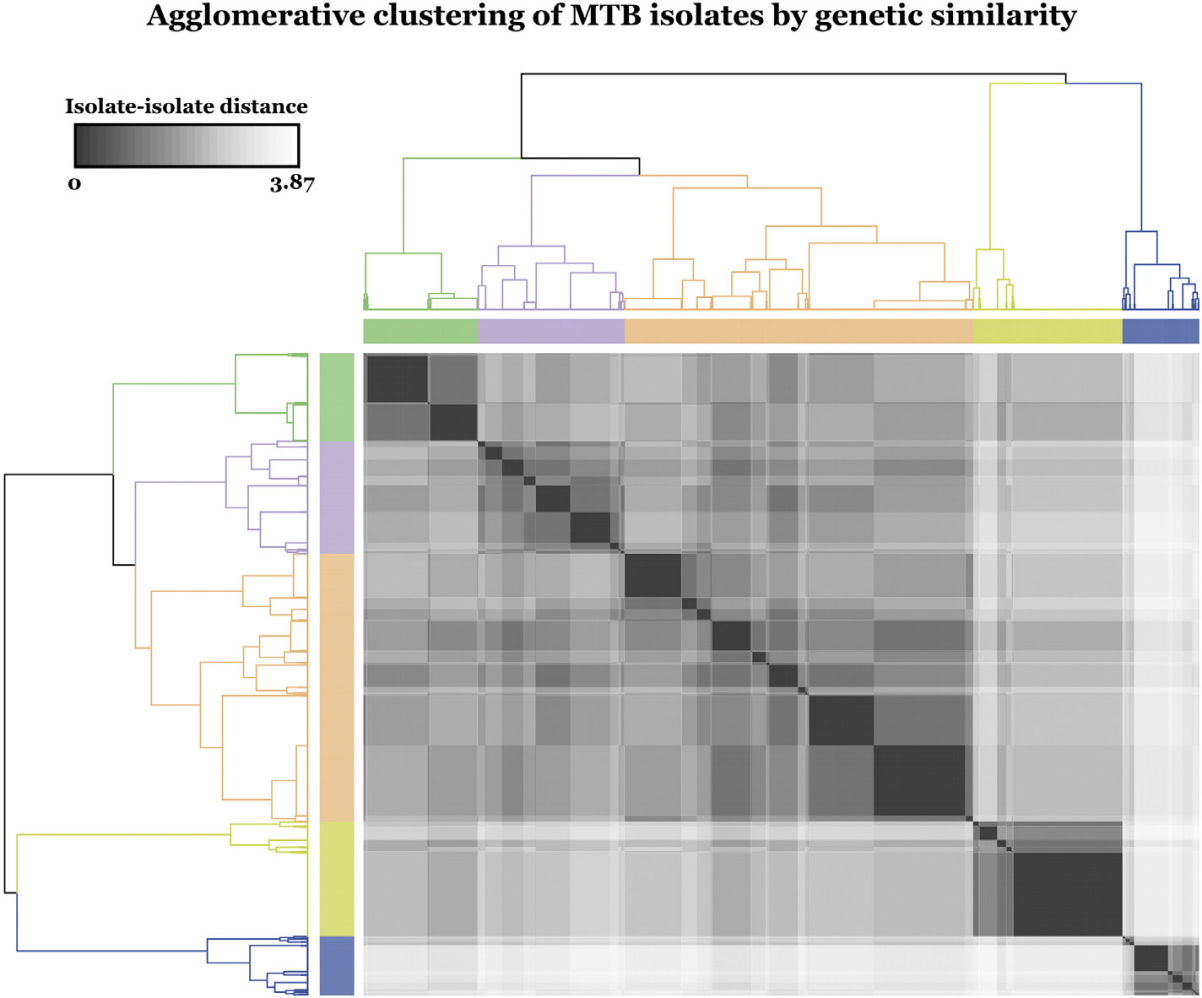
Agglomerative clustering of MTB isolates by genetic similarity. We used known lineage-defining mutations to calculate isolate-isolate Euclidean distances, which is shown in the heat map. Using these distances of the lineage-defining mutation vectors between isolates, we applied Ward's method of hierarchical clustering to construct the dendrogram and determine the five lineage clusters. The dendrogram is colored by the corresponding isolate genetic lineage. Green: East Asian, Purple: Euro-American (Latin America Mediterranean-LAM sublineage), Orange: Euro-American (other sublineages than LAM), Yellow: Central Asian, Blue: Indo-oceanic & *M. africanum*. (For interpretation of the references to colour in this figure legend, the reader is referred to the web version of this article.)

Sequence data from 33 genetic lineage markers (Table S3) were available in all 3601 isolates and were used to measure genetic distance between isolates [12]. The isolates fell into five well-defined clusters that corresponded to MTB's known genetic lineages. All 5 lineages were well represented with 632 from the Euro-American Latin America Mediterranean sub-lineage, 1501 from other Euro-American sub-lineages, 331 from the Indo-Oceanic or *Mycobacterium africanum*, 643 from the Central Asian lineage, and 494 from the East Asian lineage (Fig. 1). The input genetic data *t*-SNE coordinates also largely recapitulated the genetic clustering due to lineage (Fig. S1), illustrating that the largest genetic differences between isolates were related to lineage. On the other hand, overlying *t-*SNE coordinates for the MD-WDNN's probabilistic representation (Fig. S2) confirmed that the MD-WDNN's prediction of phenotype was not simply predicting on the basis of lineage related variation.

Compared with the training data, the independent validation dataset was geographically distinct and contained a higher proportion of East Asian lineage, 351 isolates (44%), but a lower proportion of other lineages: Euro-American Latin America Mediterranean sub-lineage (63 isolates), other Euro-American (253 isolates), Central Asian lineage (32 isolates), and 93 isolates from other lineages (Indo-Oceanic, Lineage 6, bovis and africanum).

### 3.3 Comparison of models and genetic marker feature sets

To investigate the effect of different machine learning architectures, we compared three deep learning models, including the MD-WDNN, with two simpler models, L2 regularized logistic regression and random forests. All models were trained on the full feature set of common and derived features. We found the performances across the different neural net model architectures were not significantly different in our data when trained on the full feature set (Table 1). We found the random forest model to be inferior to either L2 regularized logistic regression or any of the neural net models for three of the four first line drugs, and as a result, we did not examine this model further. The most complex neural net model, the MD-WDNN, showed the highest average performance across both first and second line drugs with an AUC of 0.953, and the highest performing simple model, L2 regularized logistic regression, showed only slightly lower performance, with an average AUC of 0.949.

**Table 1:**
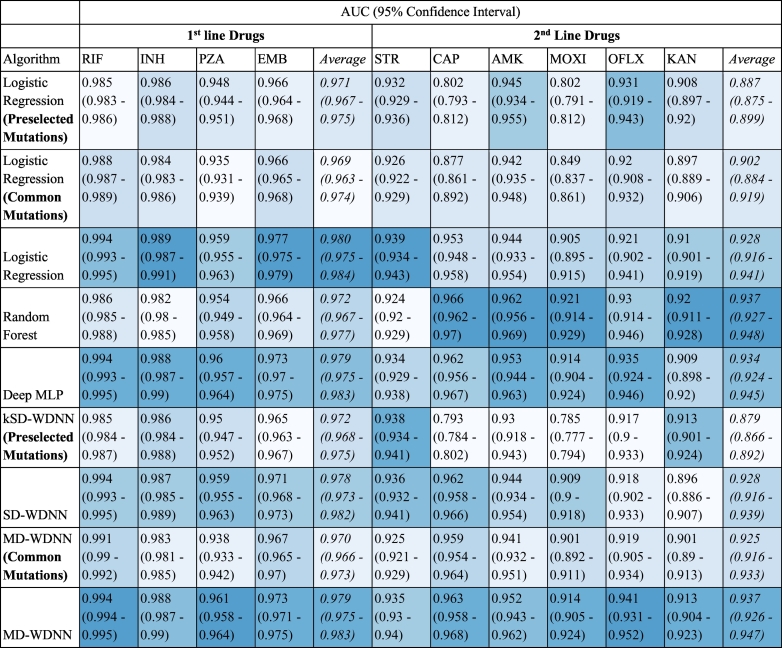
Tuberculosis drug resistance prediction AUROC performance of the models examined using repeated cross-validation. A table of predictive performance across all nine models during repeated cross-validation. The MD-WDNN, SD-WDNN, deep MLP, random forest, and logistic regression models were trained on the full set of predictors. The MD-WDNN (Common Mutations) and logistic regression (Common Mutations) models were trained on mutations not including the derived categories. The kSD-WDNN (Preselected mutations) and logistic regression (Preselected mutations) models were trained on preselected mutations known to be determinants of resistance for each drug. Performance is shown in average AUC and 95% confidence interval across all cross-validation folds. The cells are colored by rank of the model for each drug, colored from lightest to darkest corresponding with lowest to highest AUC value.

**Fig. 2:**
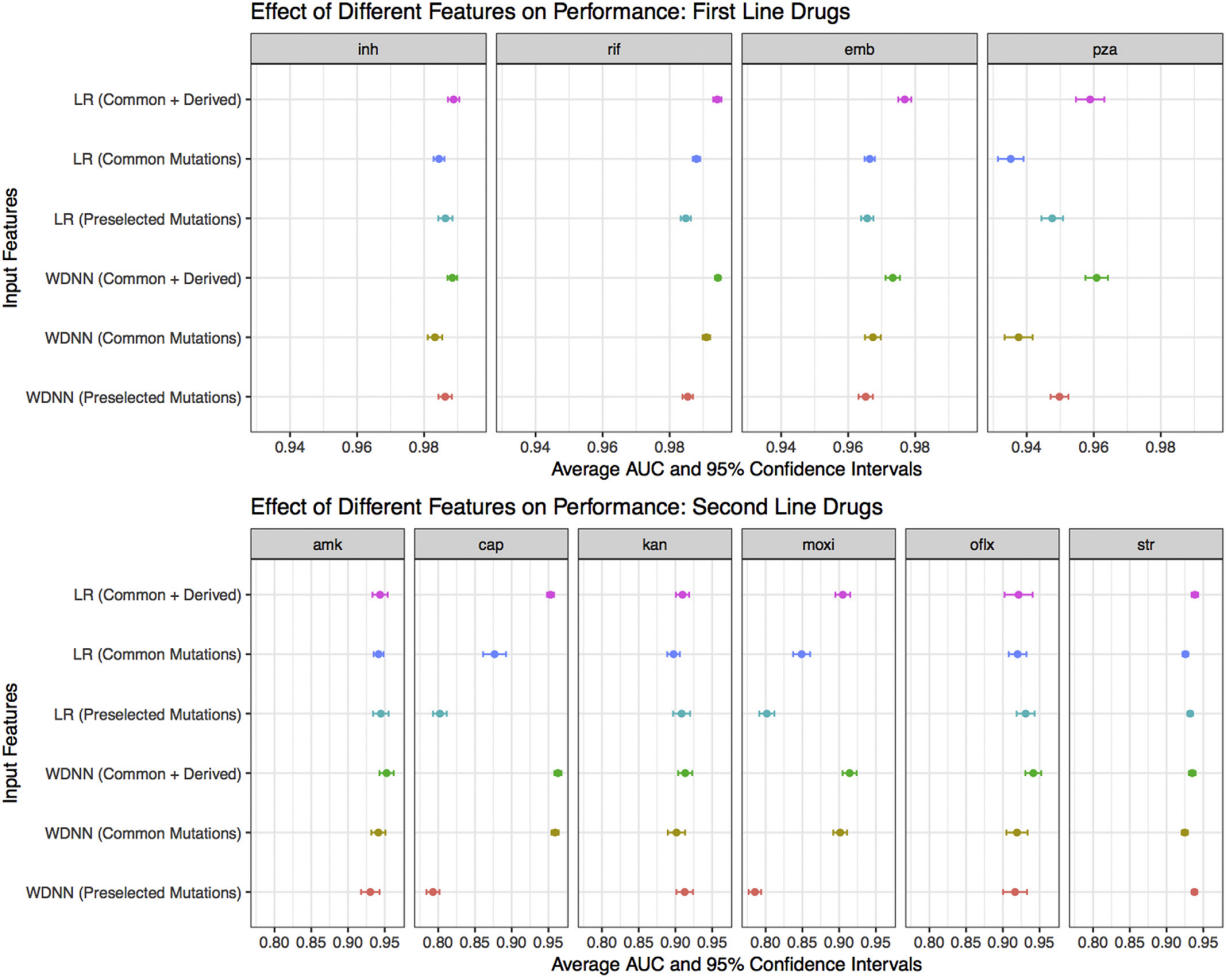
Comparison of tuberculosis drug resistance predictive performance based on input feature set. Area under the ROC curve classification performance and 95% confidence intervals during repeated cross-validation for the MD-WDNN and logistic regression models trained on all features (common and derived mutations), MD-WDNN trained on just mutations (Common Mutations), and the trained on mutations and derived categories occurring in genes known to be resistant determinants for each drug (Preselected Mutations).

To investigate the effect of different feature sets, the MD-WDNN and L2 regularized logistic regression models were trained on different subsets of genome sequence data (Fig. 2). The largest step up in AUC across any of our models and feature sets was observed between the models trained using genetic regions known to be causative of resistance for each particular drug and the models trained on the full predictor set of variants known to be determinants of resistance to at least one drug (Fig. 2, Table 1). For the second line drugs, the average AUC was 0.887 for L2 regularized logistic regression using the ‘causative’ variants *vs.* 0.929 for L2 regression using the full predictor set. The importance of using rare genetic variation in predicting resistance is highlighted by the loss of performance seen with the WDNN or L2 regression built without the derived variables (Fig. 2). This was most notable for the drugs pyrazinamide, capreomycin, and moxifloxacin.

**Fig. 3:**
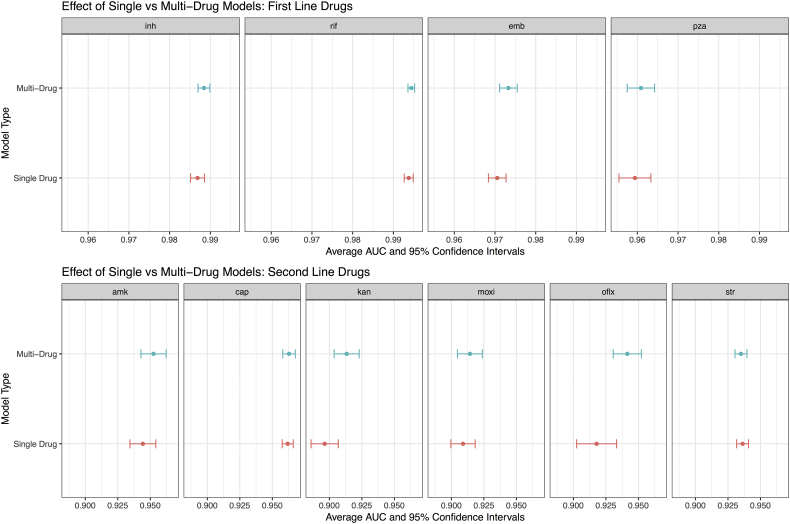
Comparison of tuberculosis drug resistance predictive performance between single drug and multidrug models. Area under the ROC curve classification performance and 95% confidence intervals during repeated cross-validation for the multidrug WDNN predicting resistance for all drugs simultaneously and for the single drug WDNN.

To compare the effect of building a single model for all drugs *vs.* individual models for each drug (*e.g.* multi-task *vs* single-task), we measured the performance of a single drug model (SD-WDNN) to the multidrug version (MD-WDNN). The predictive performance of the MD-WDNN and the SD-WDNN during repeated cross-validation are shown in Fig. 3. The average AUC for the SD-WDNN was 0.978 for first-line drugs and 0.928 for second-line drugs; the multidrug architecture of the MD-WDNN resulted in a higher average AUC for both first-line drugs (AUC = 0.979) and second-line drugs (AUC = 0.936) although these differences were not significant. The largest gains were observed for the drugs kanamycin and ofloxacin, with AUC differences of 0.023 and 0.017, respectively.

### 3.4 Independent validation

**Fig. 4:**
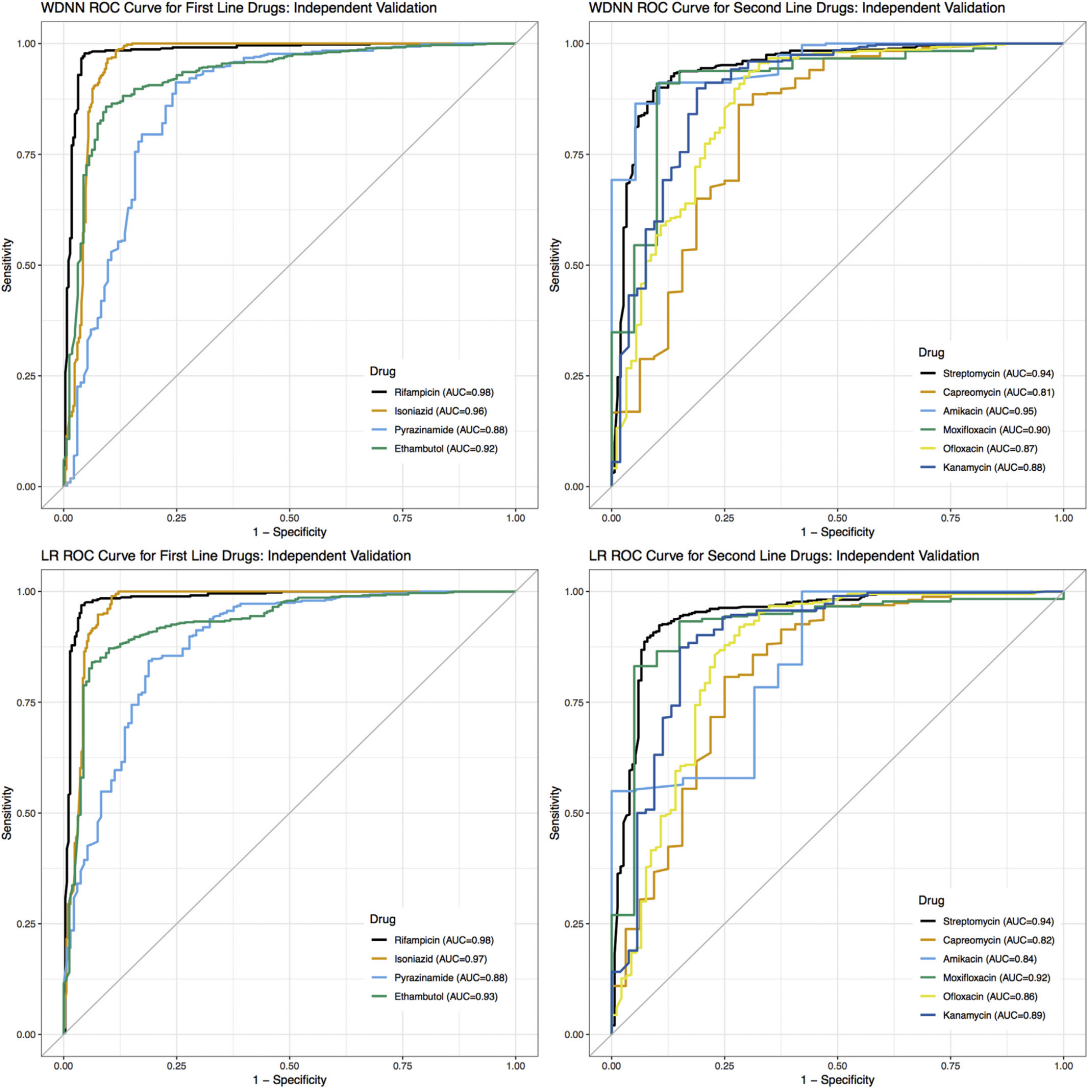
Tuberculosis drug resistance ROC performance curve of the MD-WDNN and logistic regression. A ROC plot of MD-WDNN (top) and logistic regression (bottom) predictive performance on the independent validation set for first-line (left) and second-line (right) anti-tuberculosis drugs.

The cross-validation results above supported the choice of the MD-WDNN (Average AUC = 0.953) and L2 regularized logistic regression (Average AUC = 0.949) trained on the full set of common and rare variant derived categories as high performing methods for drug resistance prediction. We proceeded to validate the model performances on an independent data set. The ROC curves for our two final models on the independent validation data across the 10 anti-tuberculosis drugs are shown in Fig. 4, illustrating the different Sn and Sp performance values at probability thresholds between 0 and 1. Table 2 shows the AUC corresponding to the ROC curves for each drug. The average AUCs for the MD-WDNN were 0.937 for first-line drugs and 0.891 for second-line drugs on an independent validation set, which were slightly lower than the AUCs during repeated cross-validation (AUC = 0.979 for first-line drugs, AUC = 0.936 for second-line drugs). The AUCs for L2 regularized logistic regression were 0.941 for first-line drugs and 0.879 for second-line drugs. Due to class imbalance for some of the drugs, we also measured performance using the precision-recall curve (Fig. S4), as this may be more informative for rare events [49]. The comparison between the MD-WDNN and logistic regression performance according to the precision-recall curve largely aligns with the AUC metric (Tables 1 and S4). We do note, however, there is a sizeable gap in average precision (AP) between MD-WDNN and logistic regression models for three drugs: capreomycin, amikacin, and moxifloxacin. The MD-WDNN achieved APs of 0.5, 0.74, 0.63 while logistic regression had APs of 0.45, 0.64, and 0.55 for those three drugs, respectively.

**Table 2:**
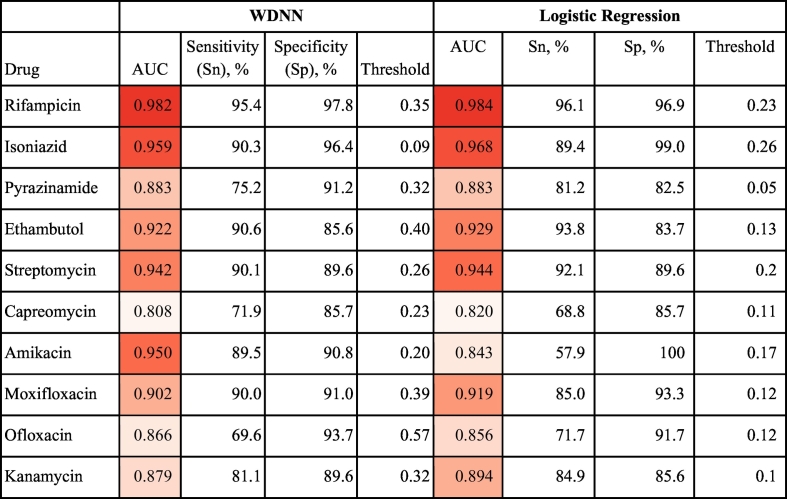
Tuberculosis drug resistance predictive performance of the MD-WDNN and logistic regression. Area under the ROC curve classification performance on the independent validation set. We also report sensitivity and specificity performance with the probability threshold chosen to maximize the sum of sensitivity and specificity for all anti-tuberculosis drugs. The cells are colored from lightest to darkest for lowest to highest AUC across the 10 drugs for each model.

Sn, Sp, and the corresponding probability threshold, which was chosen to maximize the sum of Sn and Sp, are also reported for each model and drug in Table 2. For the MD-WDNN, the average Sn and Sp, respectively, on the independent validation set were 87.9% and 92.7% for first-line drugs and 82.0% and 90.1% for second-line drugs. For L2 regularized logistic regression, the average Sn and Sp, respectively, on the independent validation set were 90.1% and 90.5% for first-line drugs and 76.7% and 91.0% for second-line drugs. Notably, the two models perform similarly, with L2 regularized logistic regression slightly higher on average, for drugs except amikacin. For amikacin, the MD-WDNN significantly outperforms L2 regularized logistic regression, with an increased AUC of 0.107 and increased sum of Sn and Sp of 22.4%. Sn and Sp values for the second probability threshold, which maximizes Sn given that Sp is at least 90%, are available in Table S5.

The prediction runtime for each model was also tested on the independent validation set. The MD-WDNN prediction time was 0.0352 s, and the L2 regularized logistic regression prediction time was 0.00291 s.

### 3.5 MTB isolate visualization using t-SNE

**Fig. 5:**
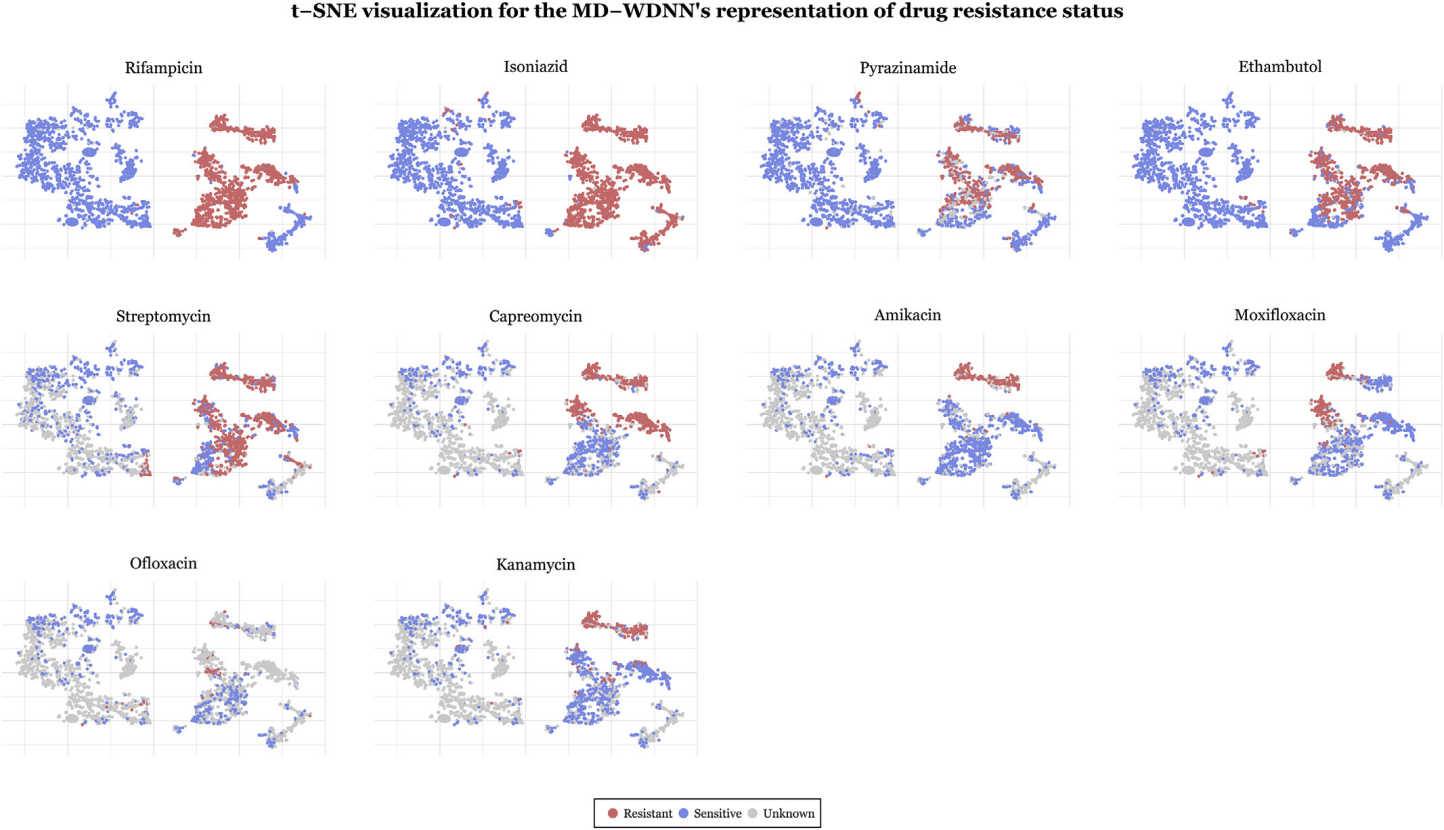
t-SNE visualization for the final output layer of the MD-WDNN. The final layer predictions, originally in 11 dimensions, were projected onto two dimensions. Each point is an MTB isolate, colored according to its resistance status with respect to the corresponding drug.

A popular way to visualize the components of a deep learning model is the *t*-distribution stochastic neighborhood embedding (*t*-SNE) method, which is a nonlinear dimensionality reduction technique [33]. We applied *t-*SNE separately to (1) the input genetic predictors and (2) the MD-WDNN predictions. *t*-SNE on the input genetic markers showed well-defined clusters, and each cluster contained both susceptible and MDR isolates with little discernable pattern of resistance classification (Fig. S3). Conversely, Fig. 5 demonstrates clear separation by the MD-WDNN's output representation between resistant and susceptible isolates, consistent with our measurements of high model Sn and Sp. The *t*-SNE plots also demonstrates the multitask WDNN's ability to classify resistance across multiple drugs, separating them into nested groups of pan-susceptible isolates, followed by mono-isoniazid resistant isolates, multidrug resistant isolates, pre-XDR isolates, and XDR isolates, which is consistent with the order of administration of the drugs clinically as well as the usual order of MTB drug resistance acquisition [34]. The second-line injectable drugs, amikacin, capreomycin, and kanamycin, also show similarly-classified clusters, highlighting the moderate level of cross resistance between them. We also observe this among the fluoroquinolones despite the fact that fewer isolates were tested for resistance to these agents [35].

### 3.6 Importance of MTB genetic variants to drug resistance

**Fig. 6:**
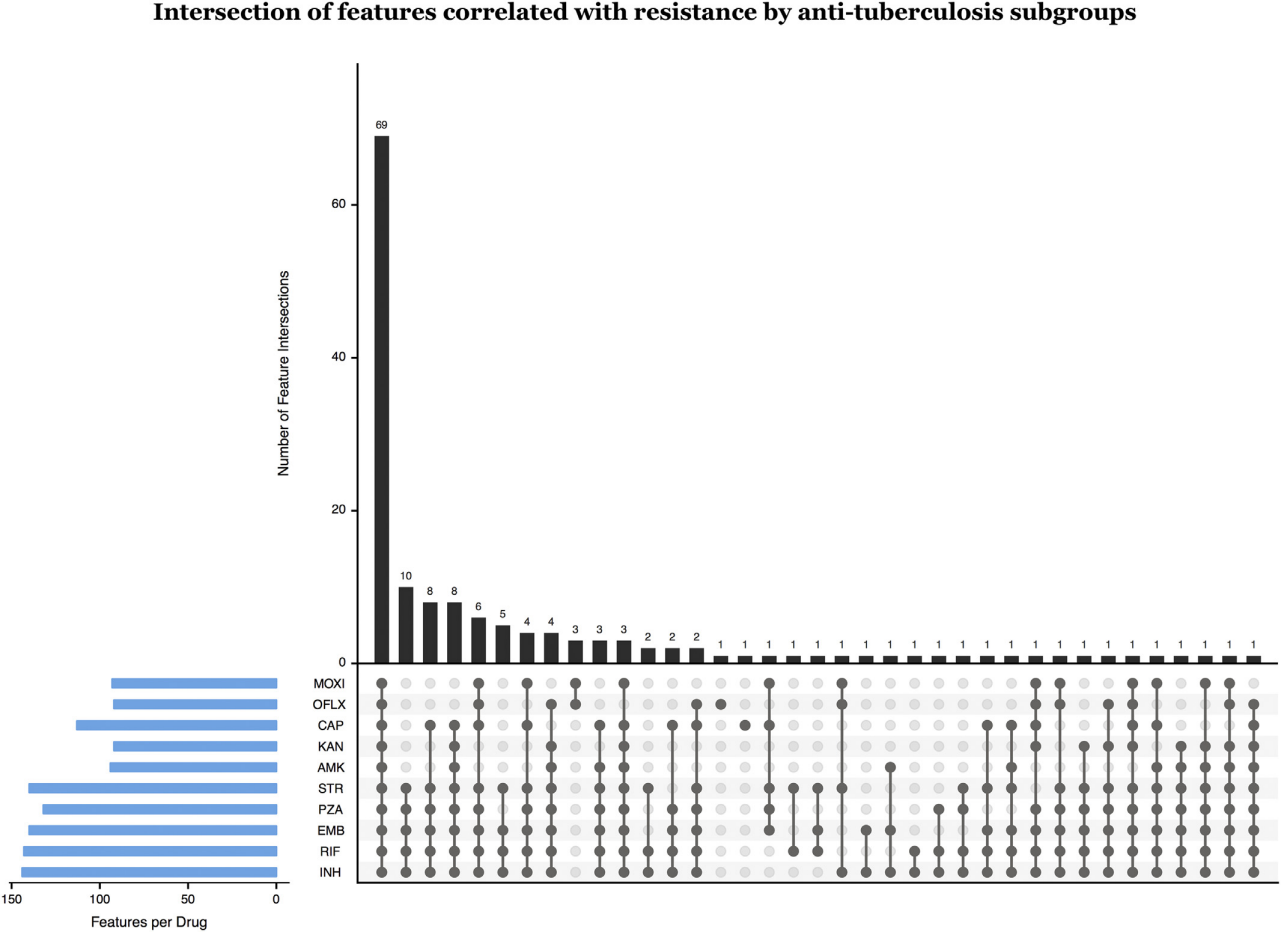
Intersection of predictors correlated with resistance by anti-tuberculosis drug subgroups. We permuted the resistance labels and calculated the distribution of the difference, P(isolate is resistant | mutation is present) – P(isolate is resistant | mutation is absent). We show the number of mutations per subgroup of drugs ordered from most to least mutations per subgroup. Number of significant predictors per drug is also shown.

All 222 predictors were tested for importance to resistance or susceptibility in the MD-WDNN to each of the 10 drugs through a permutation test as described in the methods section. Of the 156 mutations and 56 derived categories, the majority were found to be significant ‘resistance predictors’ for one or more drug: rifampicin (103 mutations, 40 derived), isoniazid (102 mutations, 42 derived), pyrazinamide (94 mutations, 38 derived), ethambutol (96 mutations, 44 derived), as well as the second-line drug streptomycin (98 mutations, 42 derived). Of the remaining predictors, the highest number of ‘susceptibility predictors’ were found in isoniazid (39 mutations, 0 derived), rifampicin (37 mutations, 1 derived), streptomycin (37 mutations, 0 derived), ethambutol (36 mutations, 0 derived), and pyrazinamide (32 mutations, 2 derived). The full list of predictors important to resistance and susceptibility in the MD-WDNN for each drug are provided in Table S6. Fig. 6 illustrates the number of significant resistance predictors per drug and their intersections among different drug subsets. Subsets of drugs that included a second line injectable drug and shared at least two predictors consistently included both INH and RIF. This is consistent with previous findings that MTB isolates acquire resistance to first-line drugs before second-line drugs [34] and indicates that the multidrug model was able to capture these relationships. The subset of fluoroquinolones shared 3 resistance-correlated predictors not found in other first-line or second-line drugs, and reflect that fluoroquinolones have a mechanism of action that differs from those of first-line and second-line drugs [36].

**Fig. 7:**
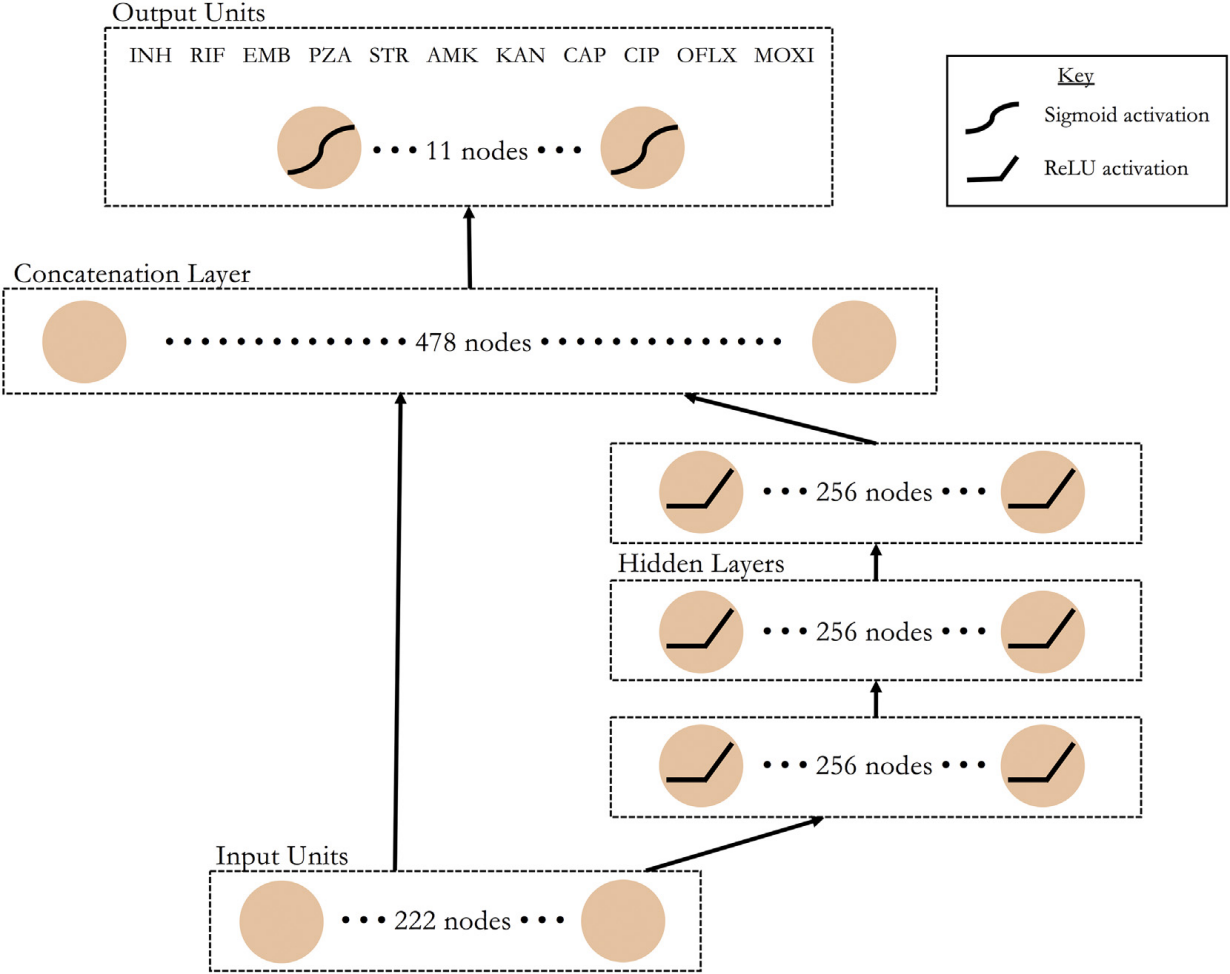
A schematic of the multidrug wide and deep neural network architecture. Data flows from bottom to top through the wide (left) and deep (right) paths of the neural network. Nonlinear transformations, where applied, are depicted on the corresponding nodes. Each of the 11 nodes in the output layer represents resistance status predictions in all MTB isolates for one of the 11 anti-tuberculosis drugs.

There was a large degree of overlap between important predictors for the MD-WDNN and L2 regularized logistic regression. The number of significant resistance predictors in overlap between the two models were 141 predictors for isoniazid, 128 for pyrazinamide, and 139 for streptomycin, including multiple derived categories. Both models successfully excluded variables known to be neutral or lineage markers, such as excluding gyrA S95 T from association with fluoroquinolone resistance. The MD-WDNN and permutation measure of importance classified a larger proportion of the variants as associated with susceptibility than did L2 regularized logistic regression. For example, 39 mutations in the MD-WDNN measure were associated with isoniazid susceptibility, whereas 2 mutations were associated with isoniazid susceptibility by L2 regularized logistic regression (Tables S6–7). Overall, 52 genetic variants were associated with susceptibility to one or more drugs, including 19 known lineage markers (Table S11). Both lists included non-canonical and rare variants among the top most important variables for resistance prediction. For example, *embA* mutations were among the top resistance predictors for the drug ethambutol with the N54D mutation as the most prominent, ranking third most important for resistance prediction. On the other hand, *embC* variants ranked much lower in importance for ethambutol, and several of its variants, including V104 M, R567H and a frameshift insertion in codon 986, were associated with susceptibility (Tables S6–7). Variants in the gene *rpsA* were individually rare but were among the top 30 predictors for the drug pyrazinamide when pooled in the derived mutation category. All predictors important for resistance and susceptibility in L2 regularized logistic regression are provided in Table S7.

## 4 Discussion

The primary aim of this study was to construct a highly accurate model of drug resistance through exploration of different machine and statistical learning methods trained on both common and rare genomic variant data. We demonstrate that L2 regression and MD-WDNN trained on a larger diverse dataset using a method of aggregating rare variants outperforms our previously reported random forest model [8]. A few prior studies have utilized algorithmic or machine learning methods using genomic data to account for the complex relationship between genotype and drug resistance in MTB [8,12,13,19,37]. Compared to one study that used a direct association (DA) algorithm, the machine learning approaches presented here offer improvement in Sn and Sp for the majority of drugs when prediction is attempted on all isolates, including those with rarer and not previously observed variants [12]. For example, DA had Sn and Sp for predicting pyrazinamide resistance of 24% and 99%, respectively, if prediction was attempted on all isolates including those with uncharacterized variants. Our MD-WDNN performance on an independent dataset achieved Sn of 75.2% and Sp of 91.2%. The best sum of Sn and Sp for the L2 regularized logistic regression model showed Sn of 81.2% and Sp of 82.5%, and fixing Sp to at least 90% for comparability with MD-WDNN results in LR Sn of 70.7%. Similarly, the WDNN and logistic regression Sn and Sp were 69.6%/93.7% and 71.7%/91.7%, respectively, for ofloxacin, whereas with DA, the Sn and Sp were 45% and 100%, respectively [12]. One study used single-task machine learning, demonstrating the validity of this approach for identifying MDR and XDR-TB, but the study did not verify their findings using independent validation data, raising concerns about generalizability [37]. Additionally, the best models in the study [37] used dimensionality reduction (sparse PCA) for two drugs (CAP and AMK) to address the problem of rare and sparse inputs, limiting the interpretability for models of these drugs. In contrast, our approach used an interpretable set of inputs (no-dimensionality reduction) for CAP and AMK, while also achieving substantially higher performance in cross-validation with AUCS of 0.96 and 0.95 for CAP and AMK, compared to AUCs of 0.85 and 0.91 reported in [37]. Across all drugs tested, our MD-WDNN approach showed higher performance in 8 of the 10 drugs during cross-validation compared to their highest performing model for each drug (Table S10). The increase in average AUC of our model was 0.014 for first-line drugs and 0.025 for second-line drugs. Third, their analysis did not demonstrate the lack of confounding by lineage and report some lineage variants as predictive of resistance.

Our approach has several novel features. First, we included all variants in the set of 32 genetic loci as potential predictors of resistance to any drug and did not subset the variants according to *a priori* knowledge of causative relationships between genetic loci and drugs. The predictive performance gains offered by this more “permissive” approach were considerable especially for the second line drugs, and the first-line “sterilizing” drug pyrazinamide [38]. Second, we utilized rare variants through our method of forming derived groups of mutations, resulting in large performance gains for certain drugs. Third, we are the first to build a neural network model for resistance prediction from MTB genotypic data. We attempted to incorporate prior information about the genetic etiology of MDR and XDR directly into the structure of our deep neural network, as it is known that both individual markers and gene-gene interactions confer resistance [[9], [10], [11]]. The wide portion of the network allows the effect of individual mutations (*e.g.* marginal effects) to be easily learned, while the deep portion of the network allows for arbitrarily complex epistatic effects to influence the predictions. Fourth, we are the first to examine a multidrug approach that allows drugs with less phenotypic data to borrow pathway information from others with a higher number of phenotyped isolates. To some extent, this proved to be true as demonstrated by Fig. 3. We acknowledge that with the use of a more complex model there is an increased risk of overfitting to our data during repeated cross-validation. We used techniques such as dropout and L2 regularization at each layer of the model to mitigate the effect of overfitting. Furthermore, we sought to evaluate potential overfitting through our analysis on an independent validation set, which showed performance with high clinical relevance. In light of these considerations, the MD-WDNN model presented here is the first multitask tool that provides the full antibiogram for 10 anti-tuberculosis drugs in one run. We successfully built high performing deep learning models to predict anti-tuberculosis drug resistance, although the performance gains from these more complex methods are not yet fully justified over simpler models, except in the case of amikacin, where the improvement was considerable. We expect the benefits of these deep learning models to increase when incorporating more genetic loci into the predictor set. Likewise, incorporating more prior information into the structure of the model, such as modeling phenomena like linkage disequilibrium using a convolutional neural network, will likely increase the performance of the deep learning approach.

Although the gains that we attribute to the multitask architecture *per se* were not significant, the gains were quantitatively larger for second line drugs like kanamycin and ofloxacin. As second line injectables and fluoroquinolones are cornerstone agents for the treatment of MDR-TB treatment, and accurate prediction of susceptibility to these agents is key in determining a patient's candidacy for the recently recommended shortened MDR-TB regimen, this approach holds promise as more genomic data is incorporated [39]. Prediction of resistance to second-line injectables has thus far been challenged by a limited genetic knowledge base and consequently limited Sn when using simple direct association approaches [12]. Thus, in aggregate, the use of a more complex approach, such as our multidrug WDNN, shows promise for performance gains in pyrazinamide and second line drugs. Furthermore, even for drugs like isoniazid and rifampicin that had high performance across the model architectures and the feature categories we tested, our multidrug WDNN validated performance exceeds prior models, achieving a respective Sn and Sp of 95% and 98% for rifampicin, and 90% and 96% for isoniazid (Table 2). Prior state-of-the-art Sn and Sp were reported at 92% and 99%, and 85% and 98%, respectively [12]. This is likely a result of using a larger and richer TB dataset that has been previously used and using a multivariate approach to prediction.

The translation of our modeling approach trained on both rare and common variants is also a function of advancements in whole genome sequencing and accessibility to more MTB isolate data. Improvements in whole-genome sequencing technologies have significantly reduced costs [40], allowing for more routine whole genome sequencing in MTB isolates [41,42]. The prediction time for MTB drug resistance depends primarily on the sequencing turnaround time, which is significantly shorter than phenotypic susceptibility testing [43]. In addition, as more routine sequencing increases the amount of MTB isolate data, our models can be rapidly updated as the datasets become accessible. We expect that as more data are incorporated, the Sn and Sp gap in second-line injectable drugs and fluoroquinolones will become smaller.

Our approach allowed us to gain new insights into the relative importance of specific genetic variants for resistance prediction. For example, the gene *rpsA* was previously causally associated with pyrazinamide resistance [25] but has not been found to be predictive of pyrazinamide resistance in clinical isolates [14]. This may relate to a low number of pyrazinamide resistant isolates that were included in prior studies or alternatively because of a more complex association between mutations in *rpsA* and the phenotype that our approach was able to capture. The protein products of the genes *embC* and *embA* are known targets of the drug ethambutol and acquisition of mutations in these genes is thought to increase ethambutol resistance levels [10,44]. Here we find several mutations in *embC* that appear to be predictors of ethambutol susceptibility and find that at least some *embA* mutations, especially N54D, to be more important resistance predictors than any of the observed *embC* mutations in our data (Tables S6–7). This is supported by recent data from genome-wide association studies of clinical isolates where *embC* variants were found to be relatively common, occurring in about 10% of isolates but nevertheless not significantly associated with ethambutol resistance [45].

We acknowledge some limitations of our study. First, one source of bias could be errors during phenotyping, as susceptibility testing for some drugs has been shown to have low reproducibility and high variance [46,47]. However, we used strains with phenotypic data measured at national or supranational TB reference laboratories following strict quality control or carefully curated from research and reference laboratories [8, 15]. Beyond technical or laboratory limitations in testing, certain resistance mutations, especially for ethambutol and second-line drugs, may result in minimum inhibitory concentrations (MICs) very close to the clinical testing concentration, which may result in lower Sn and Sp [48] when predicting a binary resistance phenotype. The use of MIC data for building future learning models may help circumvent this. Second, we only included mutations that occurred in >0.8% (30 of 3601 isolates) individually or when aggregated with other rare variants in the same gene or intergenic region. Although we may have missed some important predictors, this threshold amounted to only ignoring variants that are very rare in a diverse sample of MTB genomes with good representation from the 4 major genetic lineages. Third, we did not include third-line anti-tuberculosis drugs such as cycloserine or para-aminosalicylic acid due to the lack of phenotypic data.

In summary, we present an exploration of machine learning and traditional statistical models to identify the resistance of MTB isolates to 10 anti-tuberculosis drugs from whole genome sequencing data. Our models trained on rare and common genetic variants achieved state-of-the-art performance on a large, aggregated TB dataset, with prediction times of less than a tenth of a second, demonstrating the efficacy of our model as a diagnostic tool for MTB drug resistance. The MD-WDNN represented the first multidrug model to our knowledge that incorporated a high number of genotypic predictors known to be important to determining resistance for one or more included drugs. Further work identifying the impact of a wide range of genetic variants will not only allow for improved predictive performance but may also give us a greater understanding of the biological mechanisms underlying drug resistance in MTB isolates.

## Funding sources

This work was supported by the NIH BD2K grant K01 ES026835 and by the Bill & Melinda Gates Foundation grant OPP1115887. The funders had no role in study design, data collection, data analysis, interpretation, or writing of the report.

## Declaration of interests

Dr. Ezewudo reports grants from Bill & Melinda Gates Foundation, during the conduct of the study. Dr. Schito reports grants from Bill & Melinda Gates Foundation, during the conduct of the study. All the other authors have nothing to disclose.

## Author contributions

M.F. initially conceived the study with input from J.R.; A.D., L.F., M.S., and M.E. curated the data from public sources; M.L.C., A.B., and M.F. designed the study; M.L.C. and A.B. conducted data analyses with key input from M.F. and I.S.K.; M.L.C. initially drafted the manuscript, and all authors contributed to its final version.

## Appendix A Supplementary data

Supplementary material 1 Image 1

Supplementary material 2 Image 2

Supplementary material 2 Image 3

Supplementary material 2 Image 4
