# Supplementary material for: Beyond multidrug resistance: Leveraging rare variants with machine and statistical learning models in Mycobacterium tuberculosis resistance prediction
Source: eBioMedicine. 2019 Apr 29;43:356–69. doi: 10.1016/j.ebiom.2019.04.016 (PMC6557804; doi:10.1016/j.ebiom.2019.04.016)
Supplement: Supplementary file 1 — Supplementary material 1 [file mmc1.docx]

**Supplementary Materials**


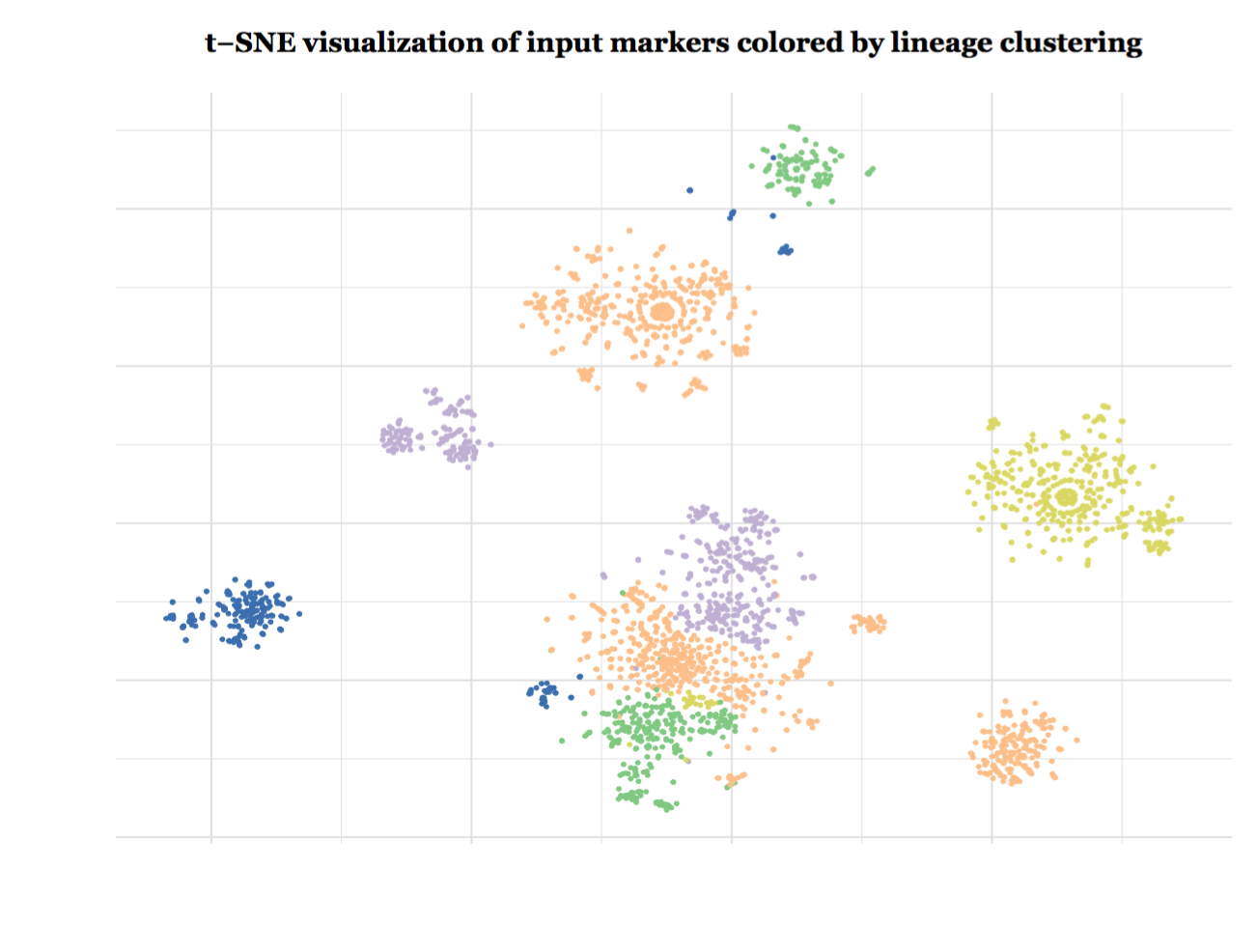


**Figure S1:** ***t*-SNE visualization for input markers colored by lineage clustering.** t-SNE plot with the same coordinates as in Figure S3. Each isolate is colored based on the five lineage clusters determined in Figure 1, illustrating that the largest genetic differences between isolates were related to lineage.

**
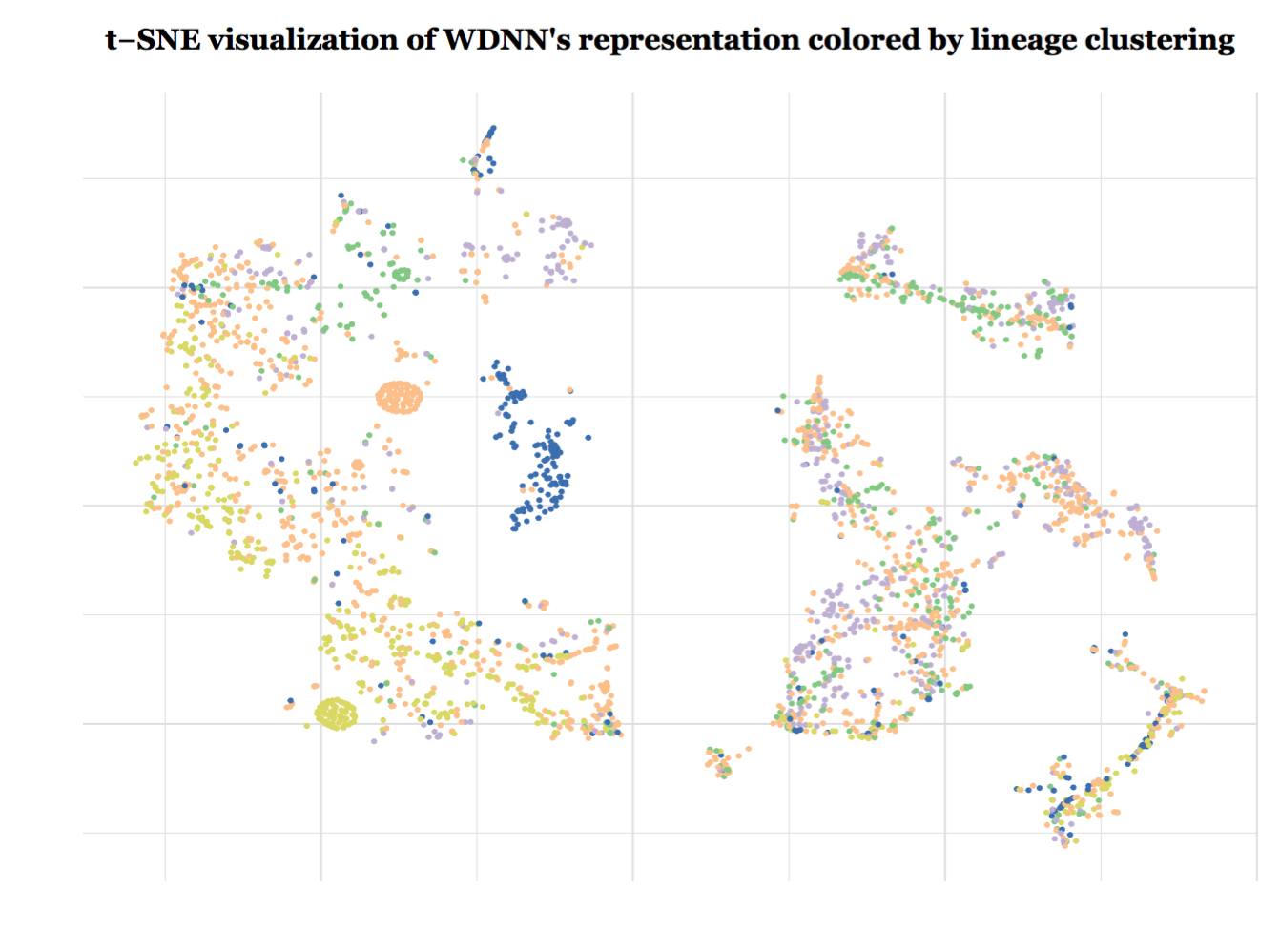
**

**Figure S2:** ***t*-SNE visualization for the final output layer of the MD-WDNN colored by lineage clustering.** t-SNE plot with the same coordinates as in Figure 5. Each isolate is colored based on the five lineage clusters determined in Figure 1, illustrating the diversity of MTB isolates within the MD-WDNN’s resistance-susceptibility clustering.


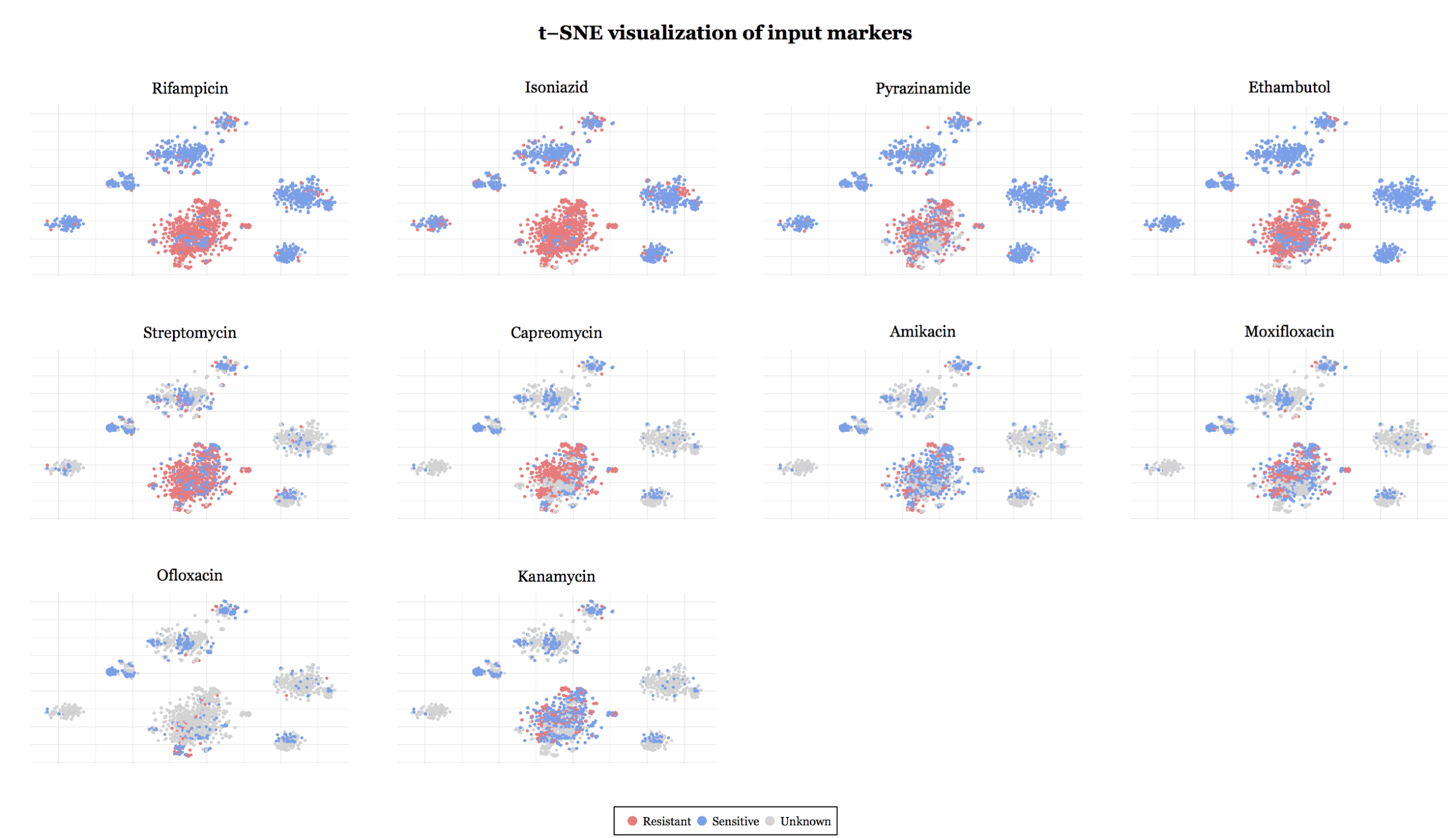


**Figure S3:** ***t*-SNE visualization for inputted genetic markers colored by resistance status for each anti-tuberculosis drug.** The input genetic markers, originally in 222 dimensions, were projected onto two dimensions. Each point is an MTB isolate, colored according to its resistance status with respect to the corresponding drug. *t*-SNE on the input genetic markers showed well-defined clusters with little discernable pattern of resistance classification between clusters.


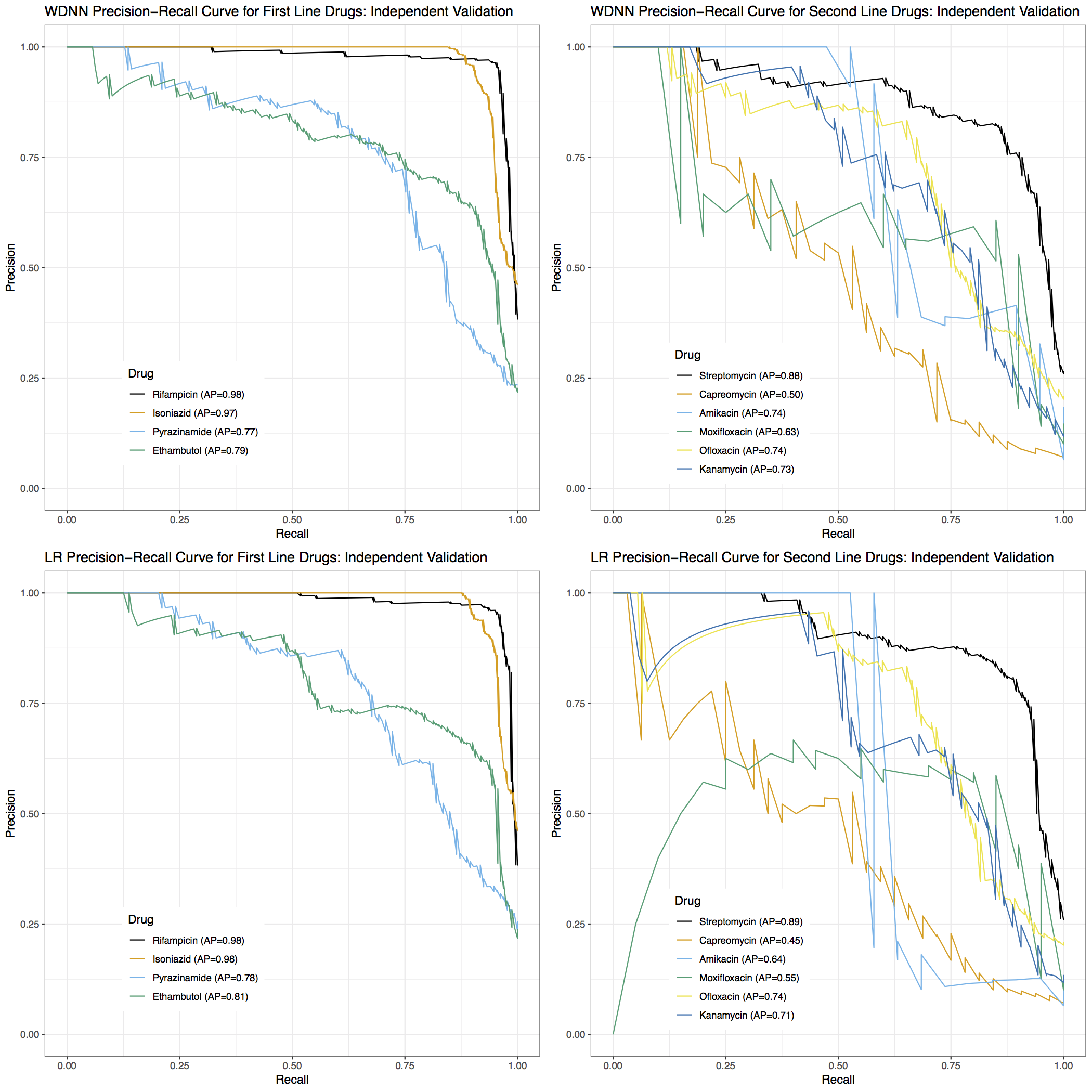


**Figure S4:** **Tuberculosis drug resistance precision-recall performance curve of the MD-WDNN and logistic regression.** A precision-recall plot of MD-WDNN (top) and logistic regression (bottom) predictive performance on the independent validation set for first-line (left) and second-line (right) anti-tuberculosis drugs.

| **Drug** | **Susceptible Isolates** | **Resistant Isolates** |
| --- | --- | --- |
| RIF | 2257 | 1285 |
| INH | 2011 | 1553 |
| PZA | 2445 | 702 |
| EMB | 2551 | 975 |
| STR | 1155 | 1025 |
| CAP | 799 | 589 |
| AMK | 1174 | 235 |
| MOXI | 1118 | 268 |
| OFLX | 651 | 88 |
| KAN | 1060 | 272 |

**Table S1**: **Phenotype of 3,601 *Mycobacterium* tuberculosis isolates in training and repeated cross-validation.** Phenotype availability for the 10 anti-tuberculosis drugs.

| **Drug** | **Susceptible Isolates** | **Resistant Isolates** |
| --- | --- | --- |
| RIF | 453 | 282 |
| INH | 384 | 330 |
| PZA | 434 | 133 |
| EMB | 576 | 160 |
| STR | 433 | 152 |
| CAP | 420 | 32 |
| AMK | 273 | 19 |
| MOXI | 178 | 20 |
| OFLX | 363 | 92 |
| KAN | 396 | 53 |

**Table S2**: **Phenotype of 792 *Mycobacterium* tuberculosis isolates in the independent validation set.**

| **Lineage-defining mutations to determine isolate diversity** |
| --- |
| inhA_V78A |
| ndh_R284W |
| ndh_V18A |
| katG_R463L |
| pncA_H57D |
| iniA_H481Q |
| embC_V104M |
| embC_T270I |
| embC_N394D |
| embC_R567H |
| embC_R738Q |
| embC_V981L |
| embA_V206M |
| embA_T608N |
| embA_P913S |
| embB_Q139H |
| embB_E378A |
| gid_A119T |
| gid_S100F |
| gid_E92D |
| gid_L16R |
| gyrB_M330I |
| gyrB_A442S |
| gyrB_C48T |
| gyrA_E21Q |
| gyrA_T80A |
| gyrA_S95T |
| gyrA_G247S |
| gyrA_A384V |
| gyrA_G668D |
| rrs_C492T |
| ahpC_G-88A |
| rpoB_C-61T |

**Table S3**: **Lineage-defining mutations to determine isolate diversity**. A table of 33 mutations used to determine isolate diversity by genetic covariance and hierarchical clustering.

|  | Average Precision (95% Confidence Interval) | | | | | | | | | | | |
| --- | --- | --- | --- | --- | --- | --- | --- | --- | --- | --- | --- | --- |
|  | **1^st^ line Drugs** | | | | | **2^nd^ Line Drugs** | | | | | | |
| Algorithm | RIF | INH | PZA | EMB | *Average* | STR | CAP | AMK | MOXI | OFLX | KAN | *Average* |
| Logistic Regression **(Preselected Mutations)** | 0.972 (0.969 - 0.975) | 0.985 (0.984 - 0.987) | 0.82 (0.807 - 0.832) | 0.898 (0.894 - 0.902) | *0.919 (0.907 - 0.931)* | 0.915 (0.91 - 0.919) | 0.771 (0.761 - 0.782) | 0.871 (0.855 - 0.887) | 0.655 (0.641 - 0.669) | 0.699 (0.672 - 0.725) | 0.793 (0.77 - 0.816) | *0.784 (0.763 - 0.805)* |
| Logistic Regression **(Common Mutations)** | 0.986 (0.985 - 0.988) | 0.984 (0.983 - 0.986) | 0.756 (0.74 - 0.772) | 0.901 (0.892 - 0.91) | *0.907 (0.891 - 0.923)* | 0.896 (0.889 - 0.904) | 0.943 (0.937 - 0.948) | 0.859 (0.838 - 0.88) | 0.767 (0.743 - 0.79) | 0.649 (0.608 - 0.691) | 0.792 (0.776 - 0.808) | *0.818 (0.791 - 0.844)* |
| Logistic Regression | 0.988 (0.986 - 0.99) | 0.988 (0.987 - 0.99) | 0.861 (0.848 - 0.874) | 0.921 (0.913 - 0.929) | *0.94 (0.926 - 0.953)* | 0.889 (0.875 - 0.903) | 0.947 (0.941 - 0.953) | 0.887 (0.868 - 0.906) | 0.8 (0.781 - 0.819) | 0.695 (0.655 - 0.734) | 0.812 (0.792 - 0.831) | *0.838 (0.812 - 0.864)* |
| Random Forest | 0.978 (0.975 - 0.98) | 0.981 (0.979 - 0.983) | 0.862 (0.85 - 0.874) | 0.903 (0.894 - 0.912) | *0.931 (0.918 - 0.944)* | 0.903 (0.895 - 0.911) | 0.961 (0.957 - 0.966) | 0.909 (0.895 - 0.924) | 0.798 (0.778 - 0.818) | 0.757 (0.716 - 0.798) | 0.836 (0.818 - 0.855) | *0.861 (0.836 - 0.886)* |
| Deep MLP | 0.989 (0.987 - 0.991) | 0.987 (0.985 - 0.989) | 0.864 (0.852 - 0.876) | 0.91 (0.902 - 0.918) | *0.937 (0.924 - 0.951)* | 0.91 (0.901 - 0.918) | 0.955 (0.95 - 0.961) | 0.894 (0.877 - 0.911) | 0.799 (0.78 - 0.818) | 0.737 (0.7 - 0.773) | 0.827 (0.812 - 0.842) | *0.854 (0.83 - 0.877)* |
| kSD-WDNN **(Preselected Mutations)** | 0.972 (0.97 - 0.975) | 0.984 (0.983 - 0.986) | 0.833 (0.824 - 0.843) | 0.895 (0.891 - 0.899) | *0.921 (0.912 - 0.931)* | 0.924 (0.919 - 0.929) | 0.754 (0.74 - 0.767) | 0.863 (0.845 - 0.882) | 0.649 (0.635 - 0.663) | 0.681 (0.651 - 0.711) | 0.811 (0.796 - 0.826) | *0.78 (0.76 - 0.801)* |
| SD-WDNN | 0.989 (0.987 - 0.992) | 0.987 (0.986 - 0.989) | 0.882 (0.87 - 0.894) | 0.911 (0.902 - 0.921) | *0.942 (0.929 - 0.956)* | 0.917 (0.91 - 0.924) | 0.96 (0.956 - 0.964) | 0.894 (0.88 - 0.908) | 0.805 (0.785 - 0.825) | 0.729 (0.695 - 0.763) | 0.829 (0.812 - 0.845) | *0.855 (0.833 - 0.878)* |
| MD-WDNN **(Common Mutations)** | 0.986 (0.984 - 0.988) | 0.984 (0.981 - 0.986) | 0.797 (0.785 - 0.809) | 0.906 (0.898 - 0.915) | *0.918 (0.905 - 0.931)* | 0.909 (0.901 - 0.916) | 0.953 (0.947 - 0.959) | 0.884 (0.865 - 0.903) | 0.785 (0.764 - 0.806) | 0.685 (0.65 - 0.721) | 0.815 (0.795 - 0.834) | *0.838 (0.814 - 0.863)* |
| MD-WDNN | 0.989 (0.987 - 0.992) | 0.988 (0.986 - 0.989) | 0.871 (0.86 - 0.882) | 0.918 (0.911 - 0.926) | *0.942 (0.93 - 0.954)* | 0.913 (0.905 - 0.921) | 0.957 (0.953 - 0.962) | 0.892 (0.877 - 0.907) | 0.803 (0.784 - 0.822) | 0.712 (0.673 - 0.75) | 0.827 (0.811 - 0.843) | *0.851 (0.827 - 0.874)* |

**Table S4**: **Tuberculosis drug resistance prediction precision-recall performance of the models examined using repeated cross-validation**. A table of average precision, which summarizes the precision-recall curve, across all nine models during repeated cross-validation. The MD-WDNN, SD-WDNN, deep MLP, random forest, and logistic regression models were trained on the full set of predictors. The MD-WDNN (Common Mutations) and logistic regression (Common Mutations) models were trained on mutations not including the derived categories. The kSD-WDNN (Preselected mutations) and logistic regression (Preselected mutations) models were trained on preselected mutations known to be determinants of resistance for each drug. Performance is shown in average precision and 95% confidence interval across all cross-validation folds.

|  | WDNN | | | Logistic Regression | | |
| --- | --- | --- | --- | --- | --- | --- |
| Drug | Sensitivity | Specificity | Threshold | Sensitivity | Specificity | Threshold |
| Rifampicin | 0.968 | 0.921 | 0.03 | 0.968 | 0.914 | 0.1 |
| Isoniazid | 0.924 | 0.906 | 0.03 | 0.9454 | 0.901 | 0.1 |
| Pyrazinamide | 0.752 | 0.901 | 0.31 | 0.707 | 0.901 | 0.11 |
| Ethambutol | 0.819 | 0.903 | 0.67 | 0.813 | 0.905 | 0.34 |
| Streptomycin | 0.895 | 0.901 | 0.27 | 0.908 | 0.903 | 0.21 |
| Capreomycin | 0.594 | 0.902 | 0.36 | 0.625 | 0.9045 | 0.14 |
| Amikacin | 0.895 | 0.908 | 0.2 | 0.579 | 0.908 | 0.1 |
| Moxifloxacin | 0.900 | 0.904 | 0.36 | 0.850 | 0.923 | 0.1 |
| Ofloxacin | 0.717 | 0.904 | 0.51 | 0.717 | 0.917 | 0.12 |
| Kanamycin | 0.792 | 0.909 | 0.33 | 0.773 | 0.907 | 0.12 |

**Table S5: Tuberculosis drug resistance maximum sensitivity with a specificity greater than 90% of the MD-WDNN and L2 regularized logistic regression on the independent validation set.** Sensitivity and specificity performance with the probability threshold chosen to maximize sensitivity such that specificity is at least 90%.

| **Gene** | **Description** | **Drug resistance association** | **ID (H37Rv)** | **Strand** | **Start** | **End** | **Length** |
| --- | --- | --- | --- | --- | --- | --- | --- |
| **promoter *ahpC*** |  | Isoniazid | - | + | 2726088 | 2726192 | 105 |
| ***ahpC*** | alkyl hydroperoxide reductase C protein | Isoniazid | Rv2428 | + | 2726193 | 2726780 | 588 |
| ***alr*** | alanine racemase | Cycloserine | Rv3423c | - | 3840194 | 3841420 | 1227 |
| ***ddl*** | D-alanine-D-alanine ligase ddlA | Cycloserine | Rv2981c | - | 3336796 | 3337917 | 1122 |
| ***embA*** | membrane indolylacetylinositol arabinosyltransferase A | [Ethambutol](http://www.tbdreamdb.com/EMB_Rv3794.html) | Rv3794 | + | 4243233 | 4246517 | 3285 |
| ***embB*** | membrane indolylacetylinositol arabinosyltransferase B | Ethambutol, Isoniazid, Rifampicin | Rv3795 | + | 4246514 | 4249810 | 3297 |
| ***embC*** | membrane indolylacetylinositol arabinosyltransferase C | Ethambutol | Rv3793 | + | 4239863 | 4243147 | 3285 |
| ***ethA*** | monooxygenase | Ethionamide | Rv3854c | - | 4326004 | 4327473 | 1470 |
| ***gidB*** | glucose-inhibited division protein B | Streptomycin | Rv3919c | - | 4407528 | 4408202 | 675 |
| ***gyrA*** | DNA gyrase subunit A | Fluoroquinolones | Rv0006 | + | 7302 | 9818 | 2517 |
| ***gyrB*** | DNA gyrase subunit B | Fluoroquinolones | Rv0005 | + | 5123 | 7267 | 2145 |
| ***inhA*** | NADH-dependent enoyl-[acyl-carrier-protein] reductase | Ethionamide, Isoniazid | Rv1484 | + | 1674202 | 1675011 | 810 |
| ***iniA*** | isoniazid inductible gene protein A | Ethambutol, Isoniazid | Rv0342 | + | 410838 | 412760 | 1923 |
| ***iniB*** | isoniazid inductible gene protein B | Ethambutol, Isoniazid | Rv0341 | + | 409362 | 410801 | 1440 |
| ***iniC*** | isoniazid inductible gene protein C | Ethambutol, Isoniazid | Rv0343 | + | 412757 | 414238 | 1482 |
| ***kasA (fabF1)*** | 3-oxoacyl-[acyl-carrier protein] synthase 1 | Isoniazid | Rv2245 | + | 2518115 | 2519365 | 1251 |
| ***katG*** | catalase-peroxidase-peroxynitritase T | Isoniazid | Rv1908c | - | 2153889 | 2156111 | 2223 |
| **promoter *mabA*** |  | Isoniazid | - | + | 1673300 | 1673439 | 140 |
| ***mabA (fabG1)*** | 3-oxoacyl-[acyl-carrier protein] reductase (mycolic acid biosynthesis protein A) | Ethionamide, Isoniazid | Rv1483 | + | 1673440 | 1674183 | 744 |
| ***ndh*** | NADH dehydrogenase | Isoniazid | Rv1854c | - | 2101651 | 2103042 | 1392 |
| ***oxyR’*** | oxidative-stress regulatory gene (pseudogene) | Isoniazid? | Rv2427Ac | - | 2725571 | 2726087 | 517 |
| ***pncA*** | pyrazinamidase/nicotinamidase | Pyrazinamide | Rv2043c | - | 2288681 | 2289241 | 561 |
| ***rpoB*** | DNA-directed RNA polymerase beta chain | Rifampicin | Rv0667 | + | 759807 | 763325 | 3519 |
| ***rpsL*** | 30S ribosomal protein S12 | Streptomycin | Rv0682 | + | 781560 | 781934 | 375 |
| ***rrl*** | ribosomal RNA 23S | Aminoglycosides | Rvnr02 | + | 1473658 | 1476795 | 3138 |
| ***rrs*** | ribosomal RNA 16S | Aminoglycosides | Rvnr01 | + | 1471846 | 1473382 | 1537 |
| ***thyA*** | thymidylate synthase | Para-aminosalicylic acid | Rv2764c | - | 3073680 | 3074471 | 792 |
| ***tlyA*** | cytotoxin\|haemolysin | Capreomycin | Rv1694 | + | 1917940 | 1918746 | 807 |
| **Promoter *eis**** |  | Kanamycin | - | - | 2715332 | 2715471 | 139 |
| ***eis**** | N-acetyltransferase | Kanamycin | Rv2416c | - | 2714124 | 2715332 | 1208 |
| ***rpsA**** | 30S ribosomal protein S1 | Pyrazinamide | Rv1630 | + | 1833542 | 1834987 | 1445 |
| **Promoter *rpsA**** |  | Pyrazinamide | - | + | 1833379 | 1833541 | 162 |

**Table S8**: **List of genomic regions used for resistance prediction.** Regions marked with (*) were not sequenced in 1,379 isolates, but are known to be associated with resistance to kanamycin and pyrazinamide. Thus, these strains were assigned a status of 0.5 for variants within these four regions. This allowed the model to learn the contribution of these regions in the remaining 2,222 isolates to antibiotic resistance.

| **MD-WDNN, MD-WDNN (Common Mutations), SD-WDNN, and kSD-WDNN** | |
| --- | --- |
| **Hyperparameter** | **Value** |
| L2 regularization | 10^-8 |
| Hidden units per layer | 256 |
| Number of hidden layers | 3 |
| Dropout | 0.5 |
| Learning rate | $e^{-9}$ |
| Optimizer | Adam |
| Epochs | 100 |
| Weight Initialization | Xavier uniform initializer |
| **Random Forest** | |
| **Hyperparameter** | **Value** |
| Number of trees | 1000 |
| Percentage of predictors to consider for best split | 20% |
| Percentage of samples to split a node | 0.2% |
| **Regularized Logistic Regression** | |
| **Hyperparameter** | **Value** |
| L2 regularization | Best penalty factor between 10^-5 and 10^5 |

**Table S9**: **Hyperparameters for the WDNN models and baseline models**. A table of hyperparameters for each model. The L2 regularization factor for logistic regression was determined using cross-validation to maximize the AUC within the 80% training data for each fold.

| Algorithm | RIF | INH | PZA | EMB | STR | CAP | AMK | MOXI | OFLX | KAN |
| --- | --- | --- | --- | --- | --- | --- | --- | --- | --- | --- |
| Kouchaki et. al | 0.9808 ± 0.0032 | 0.9789 ± 0.0038 | 0.9389 ± 0.0080 | 0.9625 ± 0.0054 | 0.9515 ± 0.0056 | 0.8546 ± 0.0202 | 0.9137 ± 0.0236 | 0.9027 ± 0.0296 | 0.9233 ± 0.0149 | 0.9249 ± 0.0293 |
| Logistic Regression | 0.994 (0.993 - 0.995) | 0.989 (0.987 - 0.991) | 0.959 (0.955 - 0.963) | 0.977 (0.975 - 0.979) | 0.939 (0.934 - 0.943) | 0.953 (0.948 - 0.958) | 0.944 (0.933 - 0.954) | 0.905 (0.895 - 0.915) | 0.921 (0.902 - 0.941) | 0.91 (0.901 - 0.919) |
| MD-WDNN | 0.994 (0.994 - 0.995) | 0.988 (0.987 - 0.99) | 0.961 (0.958 - 0.964) | 0.973 (0.971 - 0.975) | 0.935 (0.93 - 0.94) | 0.963 (0.958 - 0.968) | 0.952 (0.943 - 0.962) | 0.914 (0.905 - 0.924) | 0.941 (0.931 - 0.952) | 0.913 (0.904 - 0.923) |

**Table S10:** **Comparison of performance to prior study.** A table containing the AUCs for the best performing model in Kouchaki et al. [37] for each drug and our models’ performances during cross-validation. We show higher performance of our MD-WDNN model for 8 of the 10 drugs. For the drugs in which Kouchaki et al. used dimensionality reduction (capreomycin and amikacin), we show significantly higher performance of our MD-WDNN model.
